# VSTM2A reverses immunosuppression in colorectal cancer by antagonizing the PD-L1/PD-1 interaction

**DOI:** 10.1016/j.ymthe.2024.10.002

**Published:** 2024-10-10

**Authors:** Yujuan Dong, Jiaxun Jade Liu, Yunfei Zhou, Wei Kang, Shanglin Li, Alvin H.K. Cheung, Yi Hu, Rui Liao, Nathalie Wong, Chi Chun Wong, Simon S.M. Ng, Jun Yu

## Main text

(Molecular Therapy *32*, 4045–4057; November 2024)

In the original Figure 1 of this article, the schematic diagram of generation *Vstm2a* whole-body knockout mice was incorrect. The authors have used CRISPR/Cas9, but not Cre-LoxP system, for the deletion of exon 2-3 of *Vstm2a* gene. This error does not affect other figures or the conclusions of this paper. The authors sincerely apologize for any inconvenience the error may have caused.

**Figure:**
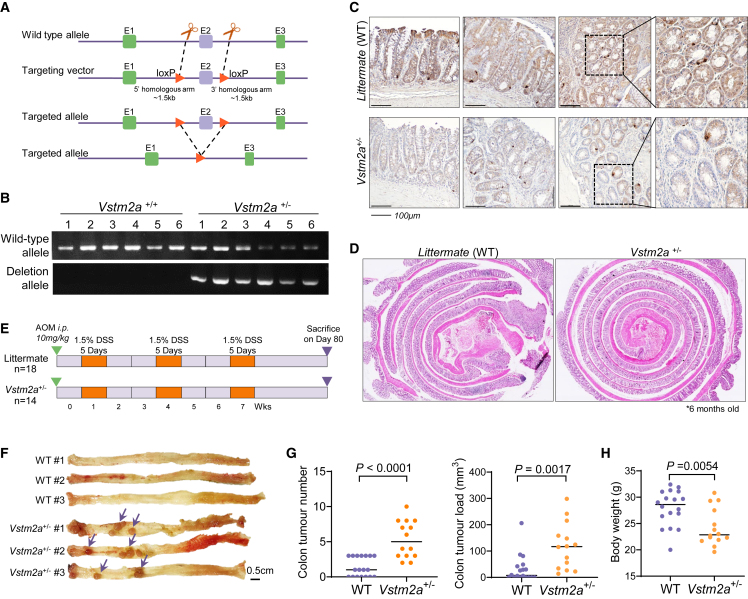
Figure 1. Heterozygous knockout of *Vstm2a* promotes CRC development in an AOM/DSS mouse model (original)

**Figure:**
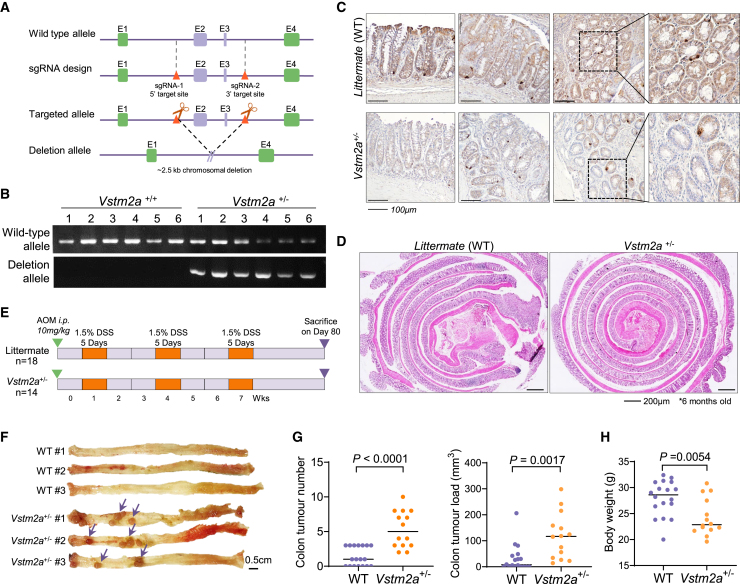
Figure 1. Heterozygous knockout of *Vstm2a* promotes CRC development in an AOM/DSS mouse model (corrected)

