# Supplementary material for: VSTM2A reverses immunosuppression in colorectal cancer by antagonizing the PD-L1/PD-1 interaction
Source: Mol Ther. 2024 Sep 17;32(11):4045–57. doi: 10.1016/j.ymthe.2024.09.023 (PMC11573612; doi:10.1016/j.ymthe.2024.09.023)
Supplement: Document S1. Figures S1–S7 and Tables S1 and S2 [file mmc1.pdf]

YMTHE, Volume *32*

Supplemental Information

VSTM2A reverses immunosuppression in colorectal cancer by antagonizing the PD-L1/PD-1 interaction

Yujuan Dong, Jiaxun Jade Liu, Yunfei Zhou, Wei Kang, Shanglin Li, Alvin H.K. Cheung, Yi Hu, Rui Liao, Nathalie Wong, Chi Chun Wong, Simon S.M. Ng, and Jun Yu

Figure S1

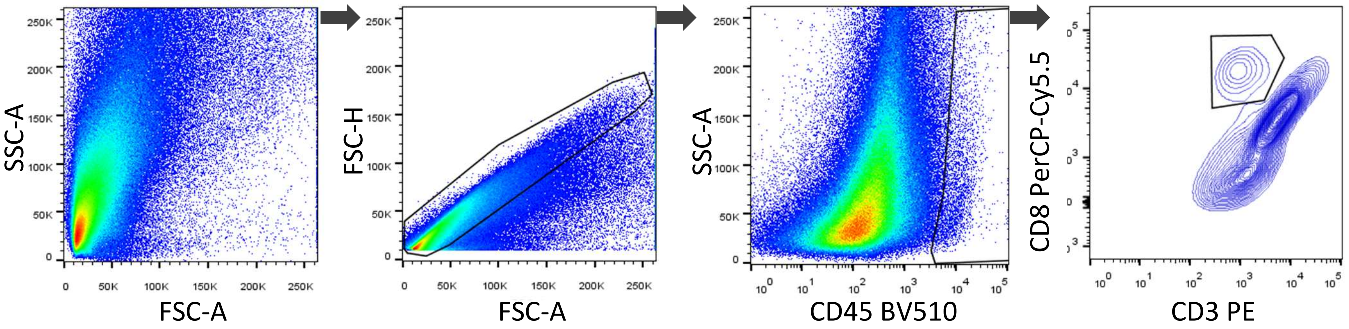

Figure S1. Gating strategy of immune cell subsets in AOM/DSS immunocompetent mice model.

Figure S2

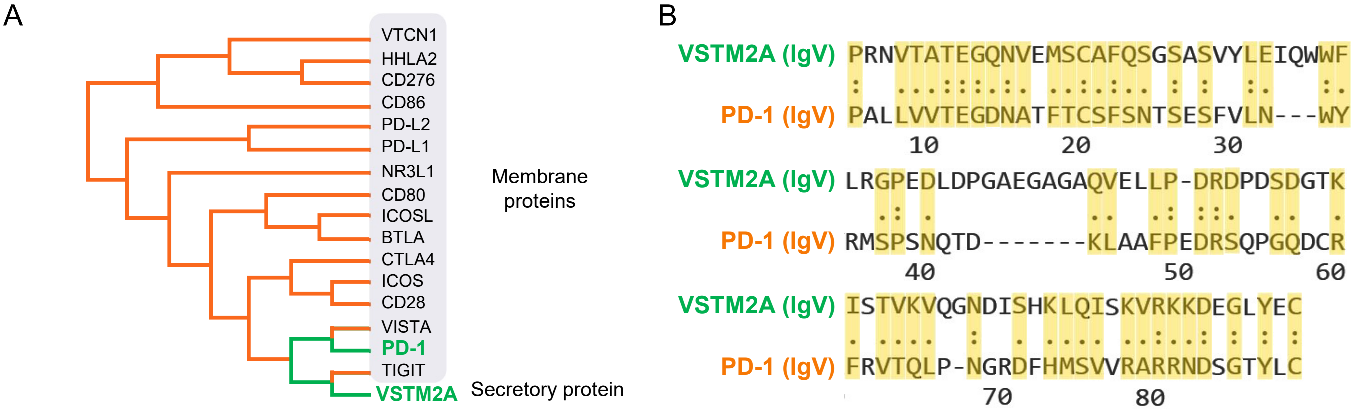

Figure S2. (A) Phylogenetic tree based on the IgV domain sequences of B7 and CD28 protein families. **(B)** Protein sequence alignments of PD-1 and VSTM2A IgV domain.

Figure S3

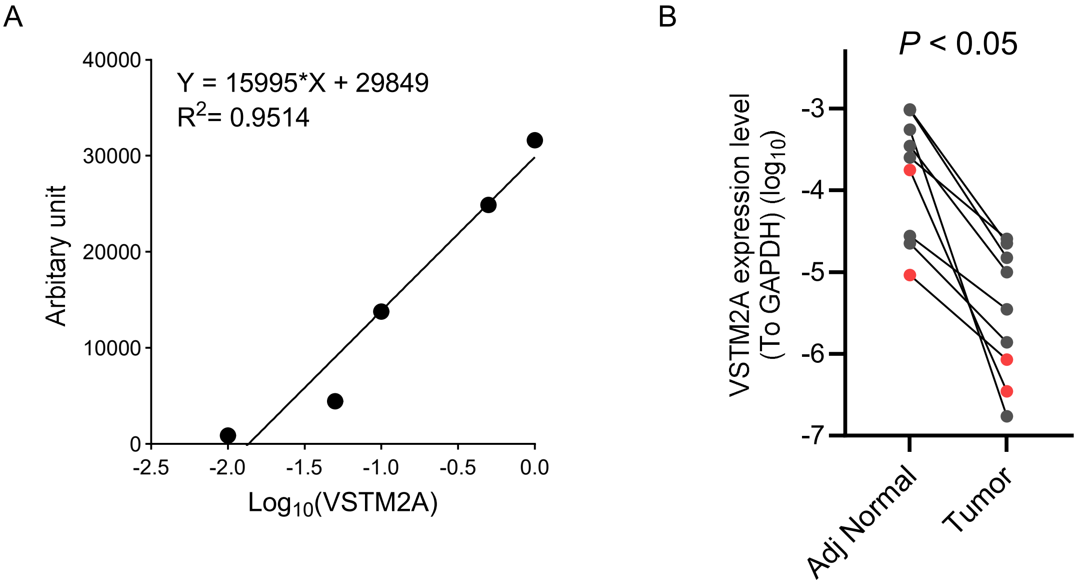

Figure S3. (A) Protein standard curve using Recombinant human VSTM2A. **(B)** VSTM2A mRNA in primary tumour and matched adjacent nontumour tissue from CRC patients were determined by QPCR (n = 9). Expression data are shown following normalization for GAPDH. Data was analyzed by paired student T test. Two cases (in red) were subjected into western blot analysis.

Figure S4

A

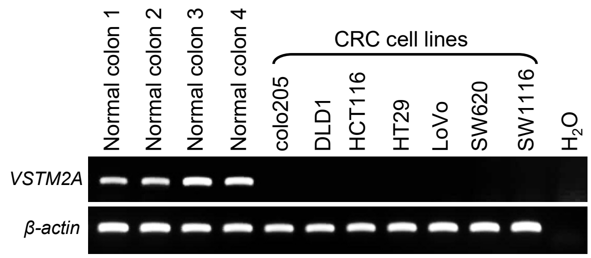

B

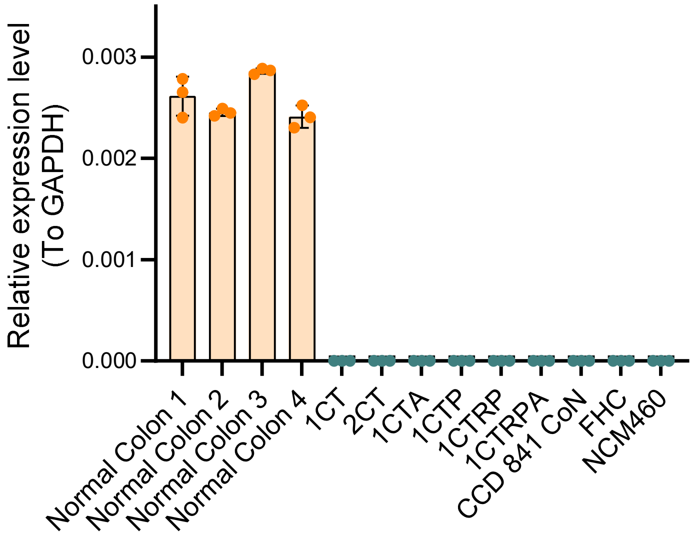

Cell line	*β-Actin (Ct)*	*VSTM2A (Ct)*
MC38	11.80	37.30
CT26	11.60	40.00
AGS	12.63	35.37
MKN28	16.03	39.40
SNU620	14.37	40.00
BGC823	16.42	40.00
MKN45	13.17	33.51
SNU638	15.49	36.84
MGC803	14.32	40.00
SNU668	13.76	37.98
TMK1	18.58	34.81

Figure S4. (A) Semi-PCR analysis of VSTM2A expression in normal colon tissues and CRC 2D cell lines. **(B)** QPCR analysis of transform and non-transformed human epithelial cells and murine CRC cell lines. Expression data are shown following normalization for GAPDH or β-Actin.

Figure S5

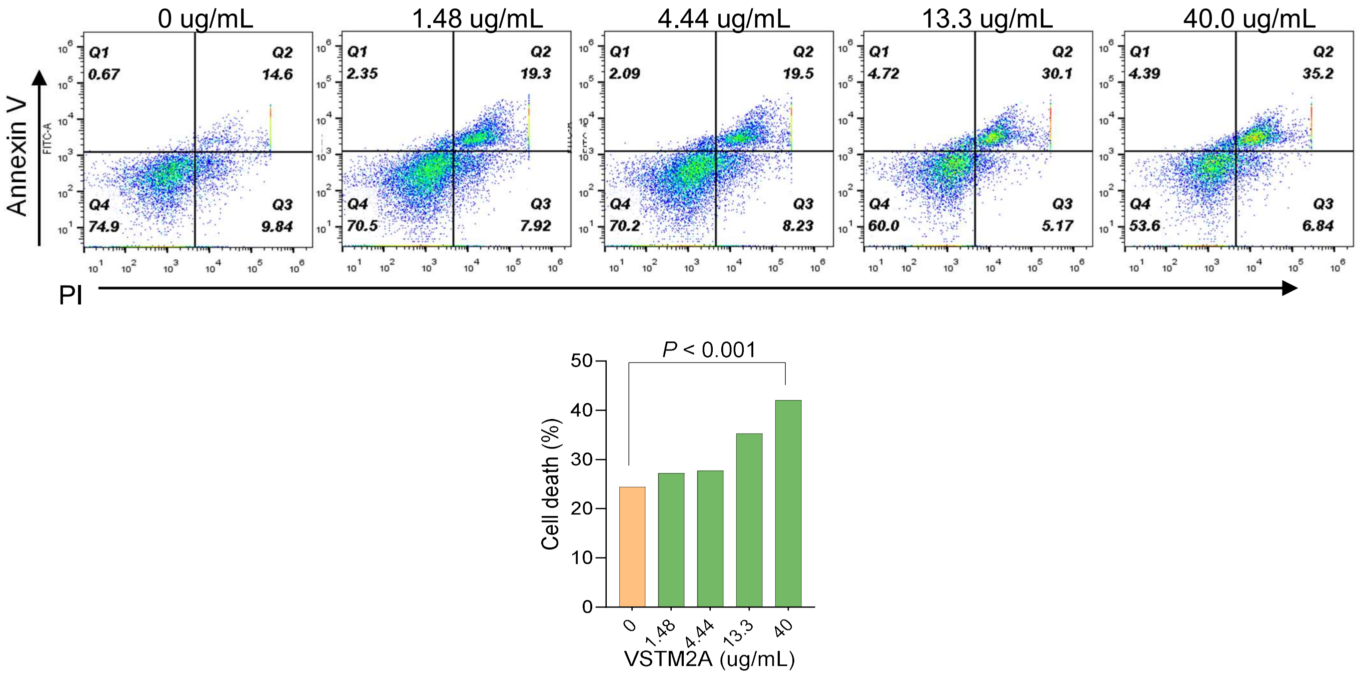

Figure S5. Cell apoptosis of PBMCs treated with various dose of recombinant human VSTM2A protein was quantified by flow cytometer.

Figure S6

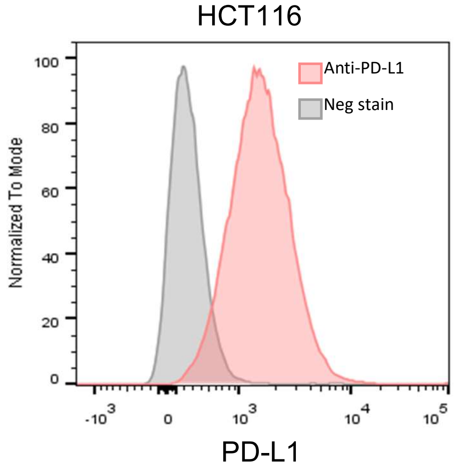

Figure S6. Cell surface expression of PD-L1 on HCT116 was determined by flow cytometry.

Figure S7

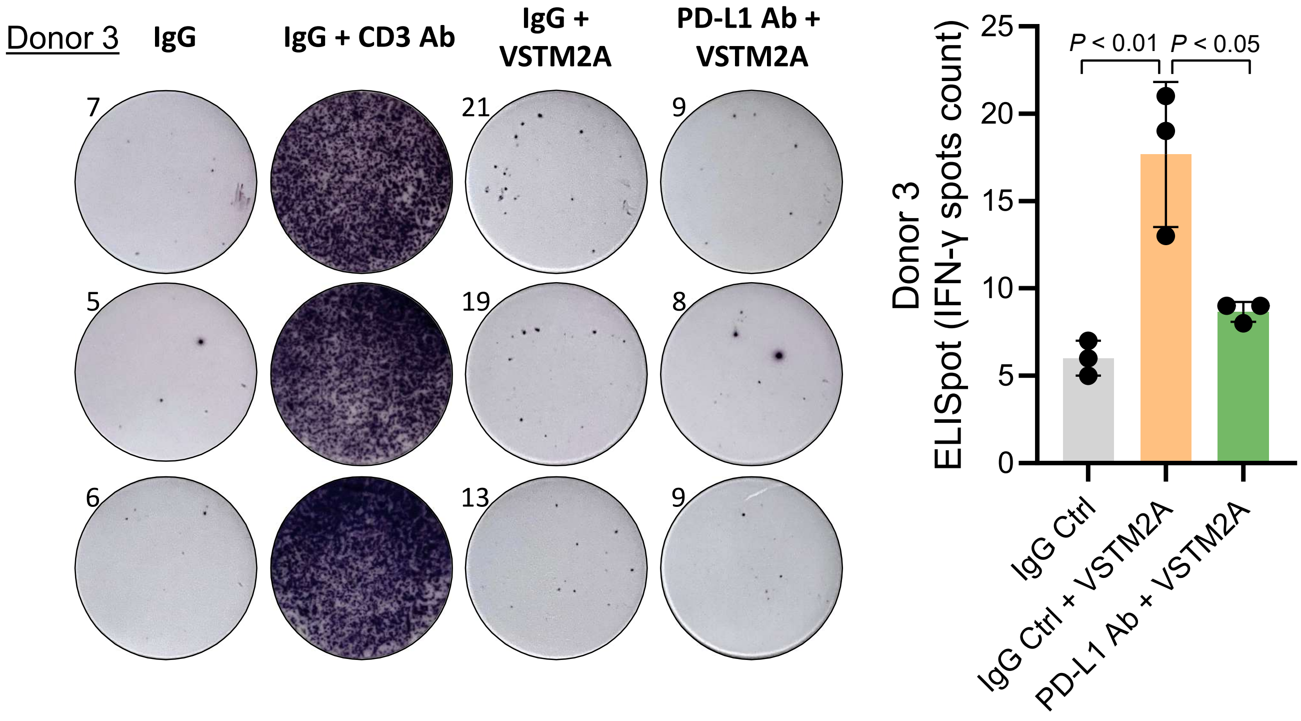

Figure S7. Images of the ELISpot assay showed the IFN-γ secretion by PBMCs (Donor 3) following 24 hours stimulation with the anti-CD3 antibody, Durvalumab (anti-PD-L1 immune checkpoint inhibitor) in the presence or absence of VSTM2A recombinant protein (1 ug/mL). Summary of the IFN-γ spot number is showed in the right panel.

Table S1. Correlation of VSTM2A expression with clinicopathologic parameters in 208 CRC patients.

**Variable**	**VSTM2A expression**				
	**Low (n=116)**	**%**	**High (n=92)**	**%**	***P*-value**
**Gender**					
Female	53	46.49	36	39.13	0.3234
Male	61	53.51	56	60.87	
**Age***					
≤60	33	28.95	28	30.43	0.8784
>60	81	71.05	64	69.57	
**AJCC stage***					
I/II	36	31.58	45	48.91	**0.0146**
III/IV	78	68.42	47	51.09	
**CD8a**					
High	37	31.90	52	56.52	**0.0004**
Low	79	68.10	40	43.48	

* 208 Case in the TMA in total with 2 cases missing of Gender, Age and AJCC stages information.

Table S2. Antibodies and QPCR primers used in this study.

Target	Supplier	Catalogue
Anti-human/mouse VSTM2A	Sigma-Aldrich	HPA024134
Anti-human GAPDH	ThermoFisher Scientific	PA585074
Anti-human PD-L1	R&D systems	AF156
Anti-human Phospho-LRP6	Cell Signaling Technology	2568P
Anti-Flag	Sigma-Aldrich	F1804
Anti-human CD8a	BioLedend	372902
Anti-human PD-L1 APC	BioLegend	329707
Anti-mouse CD8a	ThermoFisher Scientific	14008182
Anti-mouse CD45 FITC	BD Biosciences	553080
Anti-mouse CD3 PerCP-Cy5.5	BD Biosciences	560527
Anti-mouse CD45 BV510	BioLegend	103137
Anti-mouse CD8 PerCP-Cy5.5	BioLegend	100733
Anti-mouse CD3 PE	BioLegend	100206

Target	Sequence
Human-VSTM2A-QF	GGACCAAGATCAGCACAGTGAA
Human-VSTM2A-QR	TCATCCTTTTTCCTCACTTTGGA
Human-GAPDH-QF	GCACCACCAACTGCTTAGCA
Human-GAPDH-QR	TCTTCTGGGTGGCAGTGATG
Human-ACTB-QF	GTCTTCCCCTCCATCGTG
Human-ACTB-QR	AGGGTGAGGATGCCTCTCTT
Ms-Vstm2a-QF	ATGTGGCTGCAAGACACGAA
Ms-Vstm2a-QR	GGCTGGAGGTGGAGTGCAT
Ms-Actin-QF	GGCTGTATTCCCCTCCATCG
Ms-Actin-QR	CCAGTTGGTAACAATGCCATG
