# Supplementary material for: VSTM2A reverses immunosuppression in colorectal cancer by antagonizing the PD-L1/PD-1 interaction
Source: Mol Ther. 2024 Sep 17;32(11):4045–57. doi: 10.1016/j.ymthe.2024.09.023 (PMC11573612; doi:10.1016/j.ymthe.2024.09.023)
Supplement: Document S2. Article plus supplemental information [file mmc2.pdf]

Molecular Therapy
Original Article

VSTM2A reverses immunosuppression in colorectal cancer by antagonizing the PD-L1/PD-1 interaction

Yujuan Dong,[1,2,5] Jiaxun Jade Liu,[2,5] Yunfei Zhou,[1,5] Wei Kang,[3] Shanglin Li,[2] Alvin H.K. Cheung,[3] Yi Hu,[2,4] Rui Liao,[2] Nathalie Wong,[2] Chi Chun Wong,[1] Simon S.M. Ng,[1,2] and Jun Yu[1]

[1]Institute of Digestive Disease, State Key Laboratory of Digestive Disease, Department of Medicine and Therapeutics, Li Ka Shing Institute of Health Science, The Chinese University of Hong Kong, Hong Kong SAR, China; [2]Department of Surgery, The Chinese University of Hong Kong, Hong Kong SAR, China; [3]Department of Anatomical and Cellular Pathology, The Chinese University of Hong Kong, Hong Kong SAR, China; [4]Jiangxi Provincial Key Laboratory of Digestive Diseases, Department of Gastroenterology, The First Affiliated Hospital, Jiangxi Medical College, Nanchang University, Nanchang, Jiangxi, China

Immunoglobulin (Ig) VSTM2A (V-set and transmembrane domain containing 2A) is a top-ranked secretory protein frequently silenced during colorectal carcinogenesis; however, its role in immune modulation remains largely unknown. Bioinformatic and immunohistochemistry analysis of human colorectal specimens and *Vstm2a*$^{+/-}$ knockout mice indicated that VSTM2A positively correlated with CD8a and immune infiltration in both physiological and pathological conditions. We then utilized liquid chromatography-mass spectrometry to pinpoint programmed death ligand 1 (PD-L1) as a membrane receptor of VSTM2A. A series of *in vitro* biochemistry assays further revealed the binding pattern and kinetics between VSTM2A and PD-L1 proteins through their IgV domains at a dissociation constant of 0.7–2.5 nM. Recombinant VSTM2A protein inhibited the PD-1/PD-L1 interaction and induced NFAT response element (RE) luciferase activity dose dependently. Furthermore, interleukin (IL)-2 production from DO11.10 T cells upon co-culture with mouse non-T splenocytes was upregulated in the presence of VSTM2A conditioned medium. Finally, tumor killing assay and *ex vivo* data from human peripheral blood mononuclear cells and autologous dendritic cell-T cell co-culture demonstrated that VSTM2A significantly enhanced immune activation via the release of granzyme B and interferon (IFN)-γ cytokines. In conclusion, our study demonstrates the tumor-extrinsic role of VSTM2A in sterically blocking the PD-L1/PD-1 interaction at a picomole to nanomole affinity, which leads to the enhanced anti-tumor effect of cytotoxic T cells.

INTRODUCTION

Colorectal cancer (CRC) is the third most lethal and third most prevalent cancer around the globe.[1] Surgery remains the mainstay of curative treatment for CRC. However, approximately 20% of patients with CRC are diagnosed with stage IV disease, which is not amenable to curative resection. Around 25%–50% of cases will develop post-operative recurrence and metastases.[2] Targeted therapy and chemoradiotherapy remain as palliative treatments to prolong patients' survival. Unfortunately, such medical interventions have shown limited efficacy and response in patients with CRC. Therefore, it is imperative to establish novel therapeutic intervention for patients with advanced CRC.

Immune checkpoint inhibitors (ICIs) have revolutionized the treatment landscape and survival prospects of patients with advanced CRC. Initial success was achieved in the treatment of patients with unresectable or metastatic CRC with heavy gene mutation burdens (>12 per 10^6 bases) caused by mismatch-repair deficiency (dMMR) or a high level of microsatellite instability (MSI-H), which led to US Food and Drug Administration (FDA) approval of the single use of ICIs pembrolizumab and nivolumab (anti-programmed death 1 [PD-1] monoclonal antibody [mAb]) in 2017 and the combinatorial use of nivolumab and ipilimumab (anti-CTLA4 mAb) in 2018.[3–6] However, the use of humanized antibody is accompanied by low tumor penetration capacity associated with the large size of the antibody molecule (∼150 kDa), poor control of pharmacokinetics, and immune-related adverse effects.[7] Therefore, small and innate immune regulatory molecules are actively sought in this field.

V-Set and Transmembrane domain Containing 2A (VSTM2A; 26.5 kDa) is a top-ranked secretory protein that is specifically

Received 23 April 2024; accepted 13 September 2024;
https://doi.org/10.1016/j.ymthe.2024.09.023.

[5]These authors contributed equally

Correspondence: Simon S.M. Ng, Department of Surgery, Faculty of Medicine, Prince of Wales Hospital, The Chinese University of Hong Kong, Hong Kong SAR, China.
E-mail: simonng@surgery.cuhk.edu.hk
Correspondence: Jun Yu, Department of Medicine and Therapeutics, Faculty of Medicine, Prince of Wales Hospital, The Chinese University of Hong Kong, Hong Kong SAR, China.
E-mail: junyu@cuhk.edu.hk

Published by Elsevier Inc. on behalf of The American Society of Gene and Cell Therapy.
This is an open access article under the CC BY license (http://creativecommons.org/licenses/by/4.0/).

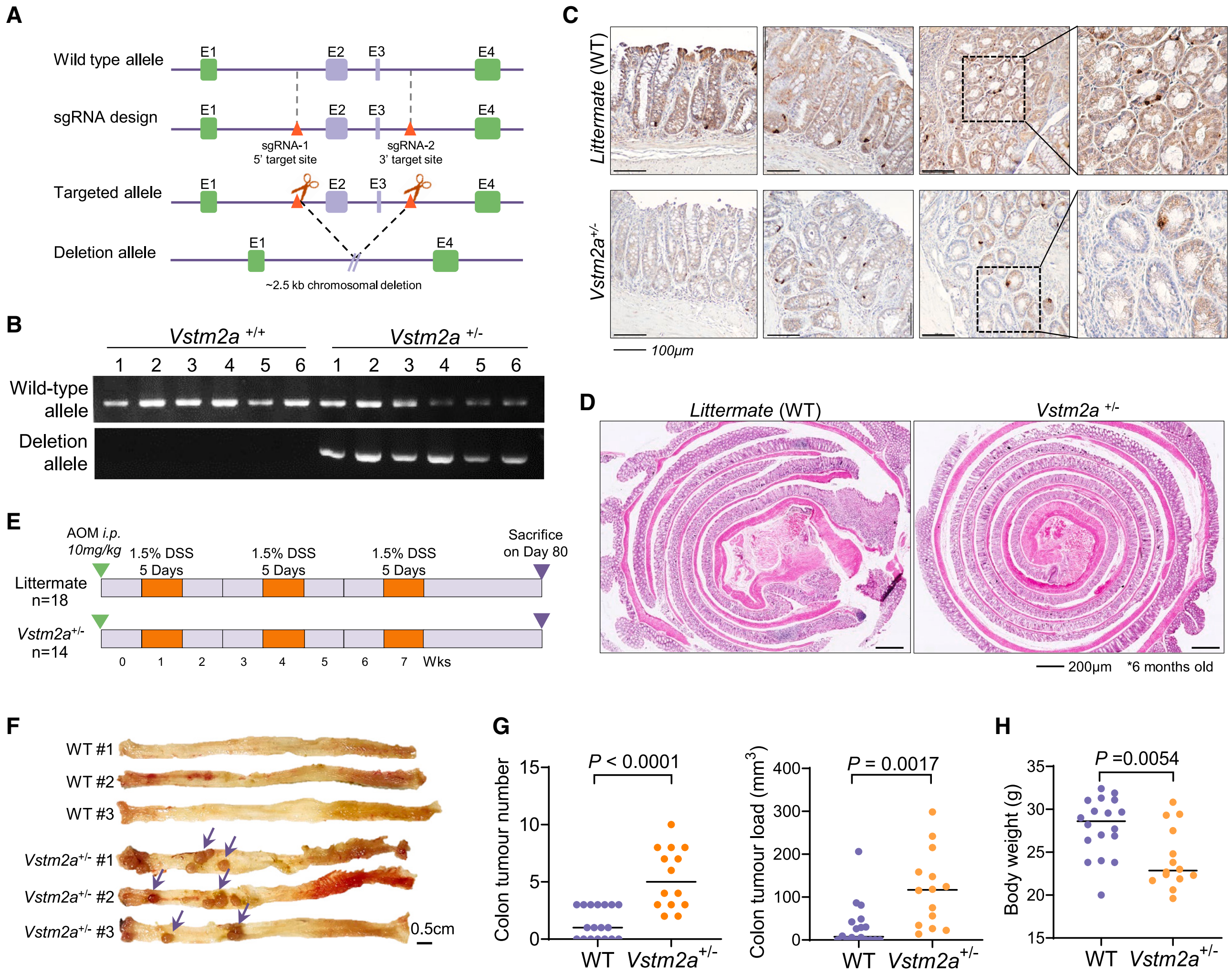

Figure 1. Heterozygous knockout of *Vstm2a* promotes CRC development in an AOM/DSS mouse model

(A) Schematic diagram of integration site of vector used to generate *Vstm2a* knockout mice. (B) Representative images of the electrophoresis patterns of amplicon products from *Vstm2a*$^{+/-}$ and wild-type mice as determined by genotyping PCR. (C) Representative image of IHC staining of Vstm2a in the colon of *Vstm2a*$^{+/-}$ knockout mice and wild-type littermates. (D) Representative image of colon histology from *Vstm2a*$^{+/-}$ mice and wild-type littermates. (E) Schematic diagram of experimental design for AOM/DSS-induced mouse CRC model. (F) Representative images of colon from *Vstm2a*$^{+/-}$ mice and wild-type littermates at the end of experiment. (G) Tumor number and tumor load in *Vstm2a*$^{+/-}$ mice and wild-type littermates. (H) Body weight of *Vstm2a*$^{+/-}$ mice and wild-type littermates at the time of sacrifice. AOM, azoxymethane; DSS, dextran sodium sulfate.

expressed in human colorectal tissue but frequently silenced by promoter methylation during CRC carcinogenesis. Our previous study reported that VSTM2A was an antagonist of Wnt ligands by competitively binding with membrane protein LRP6 and induced LRP6 endocytosis-dependent degradation, which in turn suppressed tumor-intrinsic Wnt/β-catenin signaling activation.[8] VSTM2A belongs to the immunoglobulin superfamily (IgSF) and harbors a single IgV domain. This structure is commonly present in immune receptor-ligand complexes, such as PD-1 and CD28 molecules.[9] Moreover, family members of VSTM2A, such as VSTM1 and TIGIT (VSTM3), have been demonstrated to control CD8^{+} T cell proliferation, natural killer (NK) cytotoxicity, and cytokine production.[10,11] The structural features of VSTM2A suggest its potential role in immune regulation. Therefore, in this study, we continued to elucidate the tumor-extrinsic function of VSTM2A in colorectal carcinogenesis.

RESULTS

KO of *Vstm2a* hampers CD8^{+} cell infiltration in mouse models

To further examine the role of VSTM2A in suppressing CRC carcinogenesis, we established *Vstm2a* knockout (KO) mice using Cre/*LoxP* technology (Figures 1A and 1B). No homozygous *Vstm2a* KO mice could be identified during the breeding process, suggesting that completely abolishing *Vstm2a* could induce embryonic lethality.

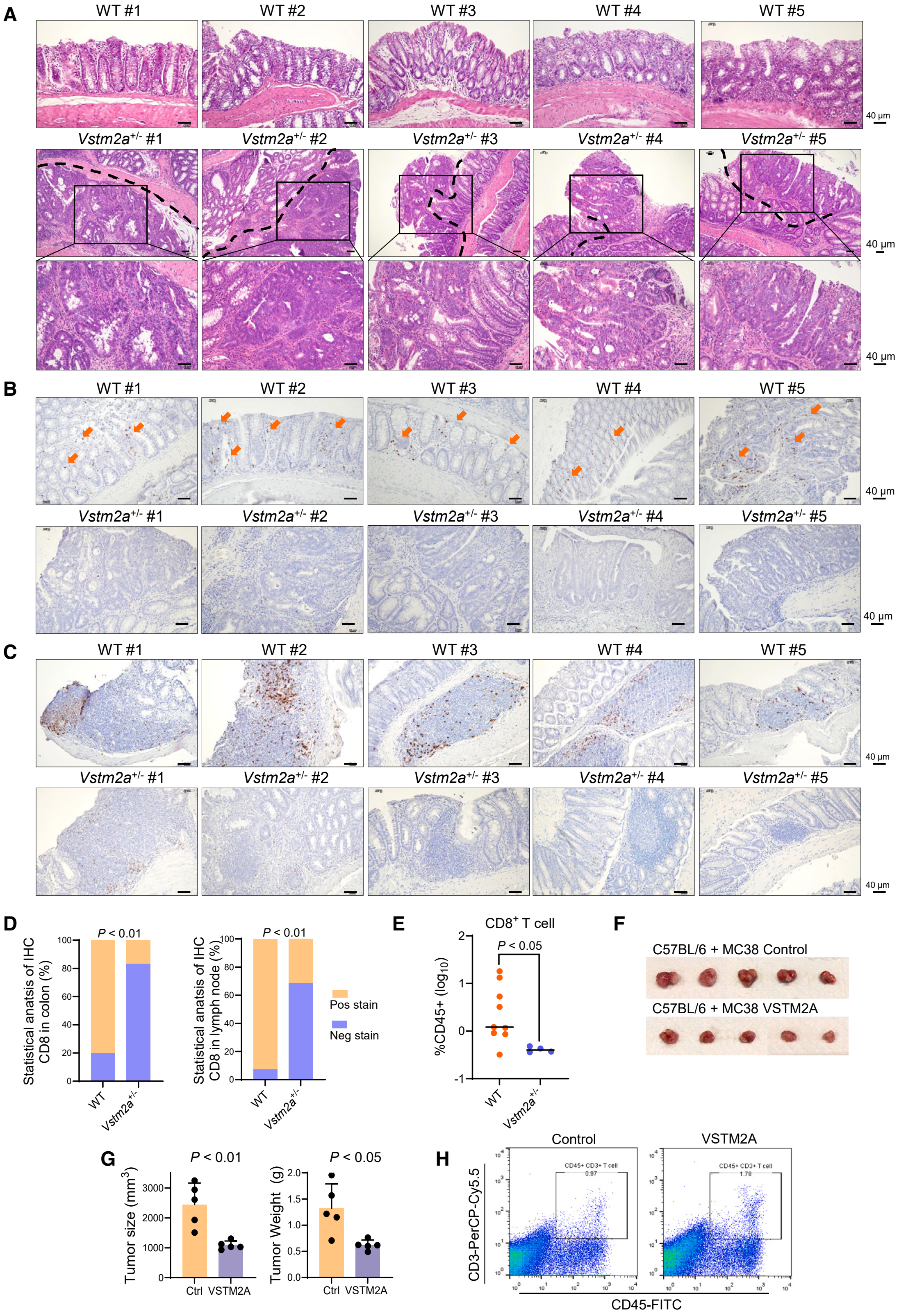

(legend on next page)

Vstm2a gene KO was assessed by immunohistochemistry (IHC) analysis (Figure 1C). Compared with the strong positive staining in the colon epithelia cells of wild-type littermates, the protein level of *Vstm2a* was significantly reduced in *Vstm2a*$^{+/-}$ heterozygous KO mice. We first conducted a 6-month period of observation on *Vstm2a*$^{+/-}$ mice and their wild-type littermates. No abnormality in the colon was found in either group, suggesting that losing one copy of *Vstm2a* will not affect colon tissue development or its normal physiological function (Figure 1D). Therefore, we challenged mice with the carcinogen azoxymethane (AOM) combined with inflammation-inducer dextran sodium sulfate (DSS) to study the function of VSTM2A in the colitis-associated CRC mice model (Figure 1E). At the time of harvest (day 80), all *Vstm2a*$^{+/-}$ mice developed visible abnormalities in colon tissue, whereas the tumor incidence was 61% in wild-type mice. Total tumor loads in *Vstm2a*$^{+/-}$ mice were significantly higher than those of wild-type mice ($p < 0.0001$ and $p < 0.01$, respectively; Figures 1F and 1G). *Vstm2a*$^{+/-}$ mice showed a significant lower body weight compared with wild-type littermates ($p < 0.01$; Figure 1H). Pathological examination of the tumors developed in *Vstm2a*$^{+/-}$ mice further confirmed high-grade dysplasia and adenocarcinoma, whereas normal colon epithelium or limited low-grade dysplasia were observed in the control group, echoing our previous report of VSTM2A as a tumor suppressor in CRC (Figure 2A). In addition to VSTM2A's tumor-intrinsic role in regulating the Wnt pathway, we further explore its tumor-extrinsic role in regulating the tumor microenvironment. In an attempt to quantify the immune cell infiltration into the tumor by IHC staining, we observed significantly less, or the absence of, CD8 signal in the dysplasia, adenocarcinoma tissues, and colon lymph nodes in *Vstm2a*$^{+/-}$ mice compared with wild-type mice, indicating that reduced *Vstm2a* could hamper T cell infiltration and immune surveillance (Figures 2B–2D). In addition, we further validated it using flow cytometry analyzing CD8^{+} T cells isolated from colon tissue in the AOM/DSS mice model. The CD8^{+} T cell population was significantly decreased in *Vstm2a*$^{+/-}$ mice colon compared with that of wild-type littermates ($p < 0.05$) (Figures 2E and S1). Conversely, a distinct increase in CD45^{+}CD3^{+} T cell infiltration was observed in syngeneic murine xenografts overexpressing human VSTM2A, further supporting the hypothesis of VSTM2A in immune regulation (Figures 2F–2H).

Silencing of VSTM2A correlates with immunosuppression in clinical CRC specimens

To further explore the significance of VSTM2A in human immune regulation, we performed IHC examinations of 208 CRC cases on a tissue microarray. In keeping with the KO mouse CRC model, we found a considerable fraction of VSTM2A-negative CRC cases with avoidance of spontaneous CD8a^{+} cell infiltration (Pearson's chi-squared test $p < 0.001$, $\phi = 0.24$, Figure 3A), indicating that silencing of VSTM2A in CRC could lead to immunosuppression. Next, we calculated the ImmuneScore of RNA sequencing (RNA-seq) datasets from The Cancer Genome Atlas (TCGA)-COAD/READ and RNA array datasets from GEO: GSE39582 using the xCell package, by which we estimated the total level of immune cell infiltration in each CRC sample.[12] Patients with inferior VSTM2A mRNA expressions showed a significantly lower ImmuneScore in both early and late stages in the two datasets, indicating a lack of VSTM2A expression associated with cold tumor environments (Figure 3B). To further evaluate the hierarchical intratumor immune cells heterogeneity, CIBERSORTx analysis was carried out in 244 TCGA-COAD cases. The median value of VSTM2A (0.027) was used as the cutoff. We observed that the T cell groups (CD4^{+} T cells, CD8^{+} T cells, gamma delta ($\gamma\delta$) T cells, and follicular helper T cells [Tfhs]) were enriched in VSTM2A-high CRC tumors (Figure 3C). In addition, we also investigated a series of normal colon RNA-seq datasets from TCGA/Genotype-Tissue Expression (GTEx) and performed Gene Ontology (GO) analysis based on the top 400 genes with a similar expression pattern to VSTM2A. Consistently, GO enrichment analysis revealed a significant enrichment of terms related to adaptive immune response and lymphocyte activation (Figure 3D), indicating that VSTM2A is positively associated with immune activation in both physiological and pathological conditions.

Secretory protein VSTM2A is a novel innate ligand of PD-L1

To investigate the mechanism of VSTM2A in regulating immune activation, we conducted a liquid chromatography-mass spectrometry (LC-MS/MS) analysis to identify membrane binding partners of VSTM2A (Figure 4A). In addition to LRP6, which has been previously identified as a receptor for VSTM2A, we found that VSTM2A interacted with the ICI programmed death ligand 1 (PD-L1) (Figure 4A). Transmembrane protein PD-L1 suppresses the adaptive immune response and facilitates tumor cells to evade immune destruction by binding with its receptor, PD-1 (also known as CD279). Given that both VSTM2A and PD-1 belong to the IgSF family and harbor one IgV domain,[9] we compared the structural features of VSTM2A and PD-1 with protein sequence clustering. Alignment indicated a 57.5% similarity and a 22.4% identity between their IgV motifs (Waterman-Eggert algorithm; Figures 4B, S2A, and S2B), suggesting that VSTM2A could function as an antagonist of the PD-L1/PD-1 interaction. To further confirm the LC-tandem MS (MS/MS) result, we performed co-immunoprecipitation (coIP) using overexpression

Figure 2. Heterozygous knockout of *Vstm2a* suppresses CD8^{+} cell infiltration

(A) Representative images of colon histology from *Vstm2a*$^{+/-}$ mice and wild-type littermates. Histological features of the high-grade dysplasia and adenocarcinoma generated from *Vstm2a*$^{+/-}$ mice are shown in the amplified images on the bottom. (B) Representative IHC images of colon tissues from *Vstm2a*$^{+/-}$ mice and wild-type littermates stained with CD8a antibody. (C) Representative IHC images of colon lymph nodules from *Vstm2a*$^{+/-}$ mice and wild-type littermates stained with CD8a antibody. (D) Statistical analysis of CD8a IHC staining in mice colon and lymph node. *Vstm2a*$^{+/-}$ mice ($n = 12$); wild-type littermates ($n = 15$). (E) Flow cytometry of CD8^{+} T cell population in *Vstm2a*$^{+/-}$ ($n = 4$) and wild-type littermates ($n = 9$). Each dot represents an independent mouse. (F) Gross tumor imaging of C57BL/6 mice subcutaneously implanted with MC38-expressing control or VSTM2A plasmid. $n = 5$ mice per group. (G) Size and weight of xenograft tumors. (H) Flow cytometry analysis of CD45^{+}CD3^{+} tumor-infiltrated T cells in xenografts expressing VSTM2A or control vector.

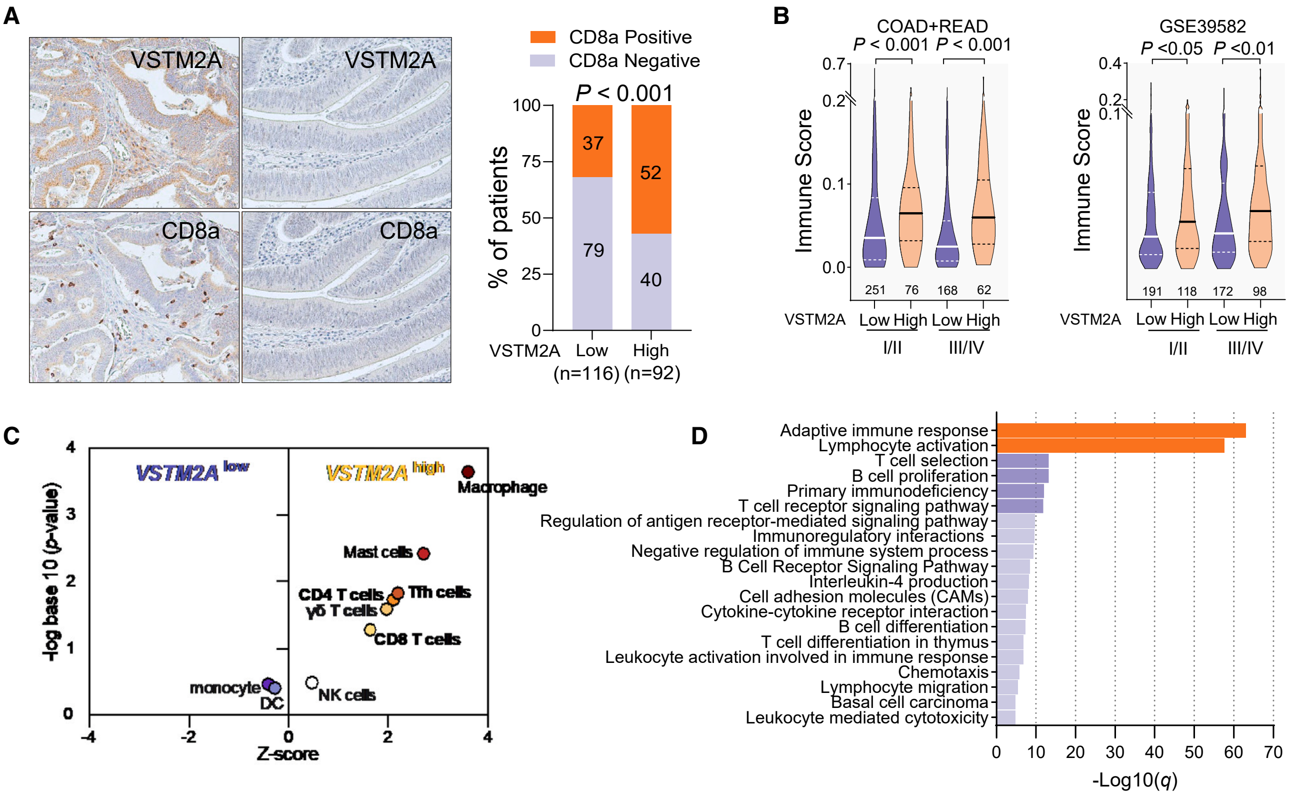

Figure 3. VSTM2A positively correlates with spontaneous immune cell infiltration in CRC

(A) Representative photomicrographs showing IHC staining of VSTM2A and CD8a in CRC samples (left). Correlation analysis of IHC staining of VSTM2A and CD8a in 208 primary CRC tissues is shown on the right. Magnification: ×200. (B) ImmuneScores were compared based on TNM stages and VSTM2A-low/-high groups in TCGA and GEO: GSE39582 datasets. The case number of each subgroup is shown in each image. (C) CIBERSORT immune fraction scores grouped by VSTM2A mRNA level. (D) GO analysis of genes positively associated with VSTM2A in colon tissue.

of both human VSTM2A and human PD-L1. As shown in Figure 4C, coIP using the enriched membrane-related protein fraction confirmed the interaction of VSTM2A and PD-L1. The interaction was further validated by reciprocal IP using VSTM2A conditioned medium and endogenous PD-L1 (Figure 4D). Mouse ortholog Pd-l1 shares a 77% amino acid (aa) sequence identity with human PD-L1.[13] In this connection, we also observed a binding between human VSTM2A and mouse Pd-l1 in the coIP assay, suggesting functional conservation of the VSTM2A/PD-L1 signal among species (Figure 4E). The extracellular domain of PD-L1 consists of IgV and IgC domains joined by a short linker.[14] To decipher the recognition pattern of the two proteins, we created a series of deletion mutations for coIP analysis. We observed that PD-L1 without IgV motif (PD-L1Δ19-127) was unable to bind with VSTM2A, whereas deletion of the IgC motif (PD-L1Δ133-225) preserved a strong interaction with VSTM2A (Figure 4F). On the other hand, the VSTM2A IgV domain (Δ144-240) was sufficient for the interaction with PD-L1 (Figure 4G). PD-L2 is a homolog of PD-L1 with 40% aa sequence identity on the IgV domain. coIP assay showed that VSTM2A could also interact with PD-L2 (Figure 4H). Taken together, our data highlight that VSTM2A is a novel ligand of PD-L1/L2 (Figure 4I). The specific binding between VSTM2A and PD-L1 is encouraging and previously undescribed in any immune research or cancer study.

VSTM2A attenuates PD-L1/PD-1 signaling and promotes T cell activity *in vitro*

To quantify the binding affinity between VSTM2A and PD-L1, we determined the protein apparent dissociation constant (K_D) using titration enzyme-linked immunosorbent assay (ELISA) with recombinant human PD-L1 Fc chimera protein mobilized on a hydrophilic protein-binding plate and gradually increasing the recombinant human VSTM2A His-tag protein. Calculated apparent K_D values ranged from 700 pM to 2.5 nM, suggesting that VSTM2A is a potent intrinsic PD-L1 ligand with high affinity (Figure 5A). PD-1 interacts with PD-L1 through the residues dispersed on the PD-1 IgV domain. Using a PD-L1/PD-1 inhibitor screening ELISA assay, we observed that recombinant VSTM2A dose dependently suppressed the interaction of PD-L1 and PD-1 with an inhibitory concentration (IC_{50}) of 4.51 nM, indicating that VSTM2A may compete with PD-1 on the same PD-L1 epitope (Figure 5B). To investigate the biological consequence of VSTM2A/PD-L1 interaction *in vitro*, we conducted a bioluminescent cell-based PD-1/PD-L1 blockade bioassay, where CHO-K1

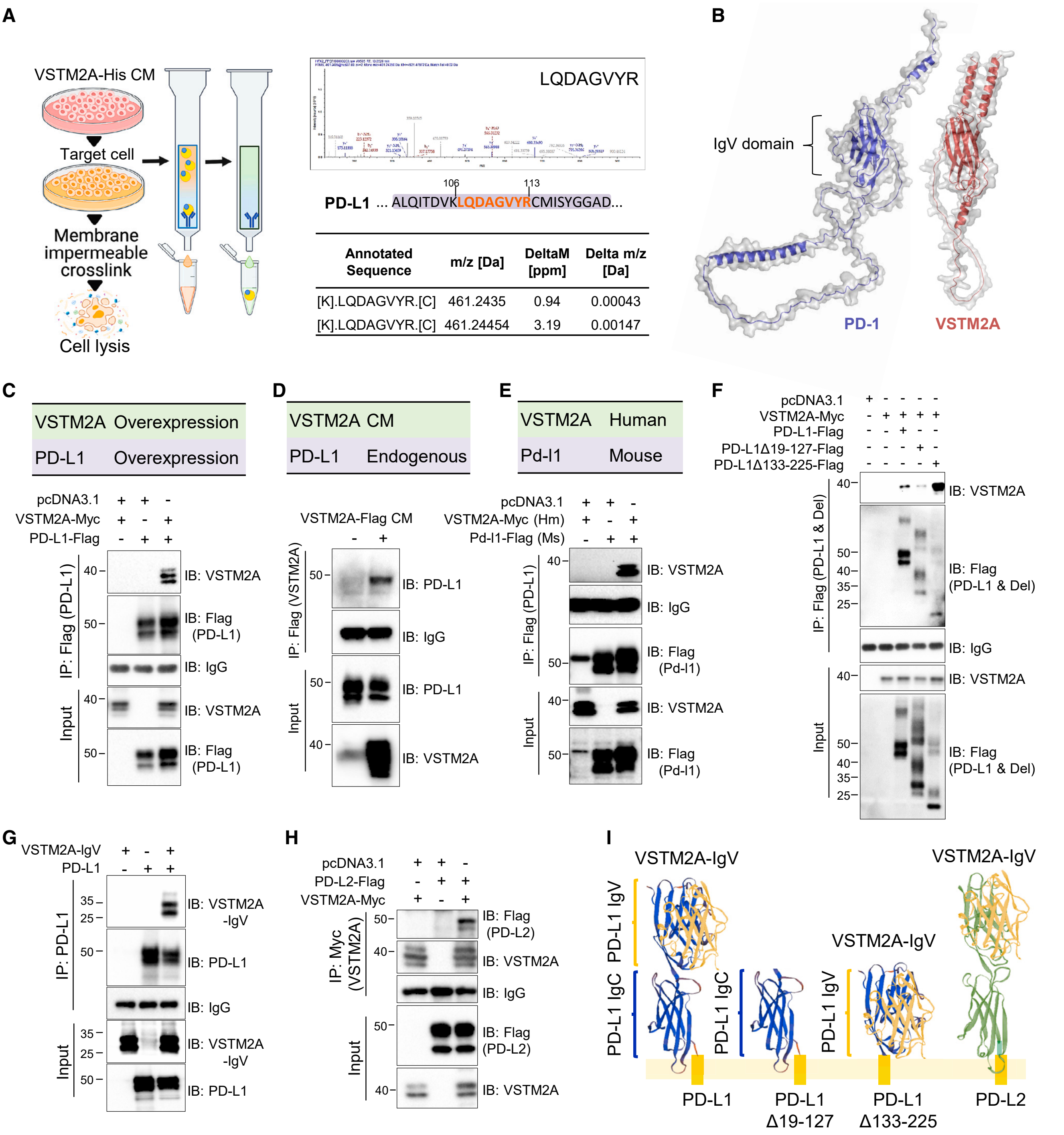

Figure 4. Identification of secretory protein VSTM2A as a novel ligand of PD-L1

(A) Experimental strategy for identification of the potential cell membrane receptor of VSTM2A by Ni-NTA purification and LC-MS/MS. MS/MS spectra of the peptides matched with human PD-L1 are shown on the right. (B) Predicted 3D structure of human VSTM2A and PD-1. (C) Detection of the interaction between human PD-L1 and VSTM2A using overexpression followed by coIP. (D) Detection of human PD-L1/VSTM2A interaction by reciprocal coIP using VSTM2A conditioned medium and endogenous PD-L1. (E) Detection of the interaction between human VSTM2A and mouse Pd-l1 by coIP. (F) Domain mapping of PD-L1 for VSTM2A binding. (G) Domain mapping of VSTM2A for PD-L1 binding. (H) Detection of the interaction between human PD-L2 and VSTM2A by coIP. (I) Summary of the interaction properties of VSTM2A with PD-L1/L2. CM, conditioned medium; Hm, human; Ms, mouse.

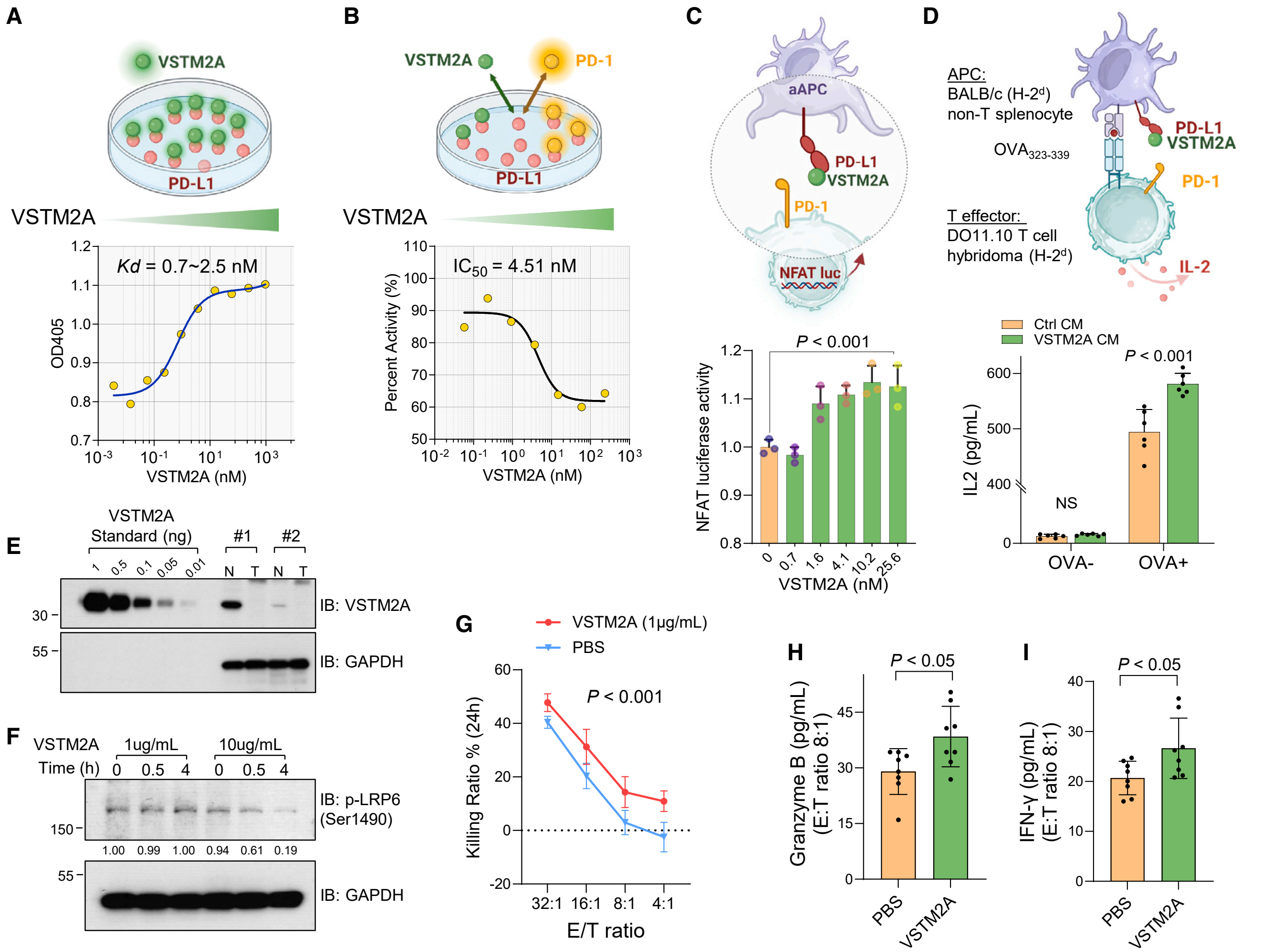

Figure 5. VSTM2A attenuates PD-L1/PD-1 signaling and promotes T cell activity

(A) Determine the dissociation constant of human VSTM2A and PD-L1 using titration ELISA. (B) Determine VSTM2A IC_{50} using PD-L1/PD-1 inhibitor screening ELISA assay. (C) Recombinant VSTM2A protein induced NFAT-RE luciferase activity dose dependently in PD-1/PD-L1 blockade bioassay. (D) IL-2 production from DO11.10 T cells upon co-culture with APCs in the presence of VSTM2A conditioned medium or control medium. (E) VSTM2A protein level in human CRC and adjacent normal tissue was determined by western blot. 20 μg total protein was loaded per lane. Recombinant VSTM2A protein was as a standard curve (0.01–1 ng was loaded per lane). (F) Human T cells were stimulated with 1 or 10 μg/mL recombinant VSTM2A protein for 30 min and 4 h. Phosphorylation of LRP6 (Ser1490) was evaluated using western blot. (G) Killing assays showing the percentage of cytotoxicity of wild-type T cells against HCT116 (a PD-L1-positive CRC cell line) in the presence of 1 μg/mL VSTM2A or PBS for 24 h. The data are shown as the mean ± SD. The experiment was repeated three independent times. Two-way ANOVA with Tukey's multiple comparisons test was used. (H) The secretion of granzyme B and (I) IFN-γ from the co-culture supernatant with HCT116 cells in the killing assay was determined using ELISA. APC, antigen-presenting cell; CM, conditioned medium; N, adjacent normal; T, tumor; E, effector; T, target cell.

cells expressing PD-L1 are antigen-independent artificial antigen-presenting cells (aAPCs) and Jurkat T cells expressing PD-1 with NFAT response element (RE)-mediated luciferase reporter are effector cells. Recombinant VSTM2A protein (700 pM–25.6 nM) significantly blocked the interaction of PD-1/PD-L1 and induced NFAT-RE luciferase activity dose dependently (Figure 5C). Furthermore, we used non-T cell spherocytes isolated from BALB/c mice as APCs to stimulate DO11.10 T hybridoma cells that recognize major histocompatibility complex (MHC) class II-restricted peptide $OVA_{323-339}$/I-Ad. Consistent with the luciferase assay, upon antigen stimulation, interleukin (IL)-2 production from DO11.10 T hybridoma cells was further enhanced in the presence of VSTM2A (Figure 5D), indicating that the VSTM2A/PD-L1 interaction inhibits PD-L1/PD-1-induced immunosuppressive signaling.

VSTM2A promotes cytotoxicity of T cells and immune activation of human PBMCs

To mimic the of role of VSTM2A in antagonizing PD-L1 signaling under physiological levels, we first evaluated the protein amount of VSTM2A in human CRC and adjacent non-tumoral tissues using

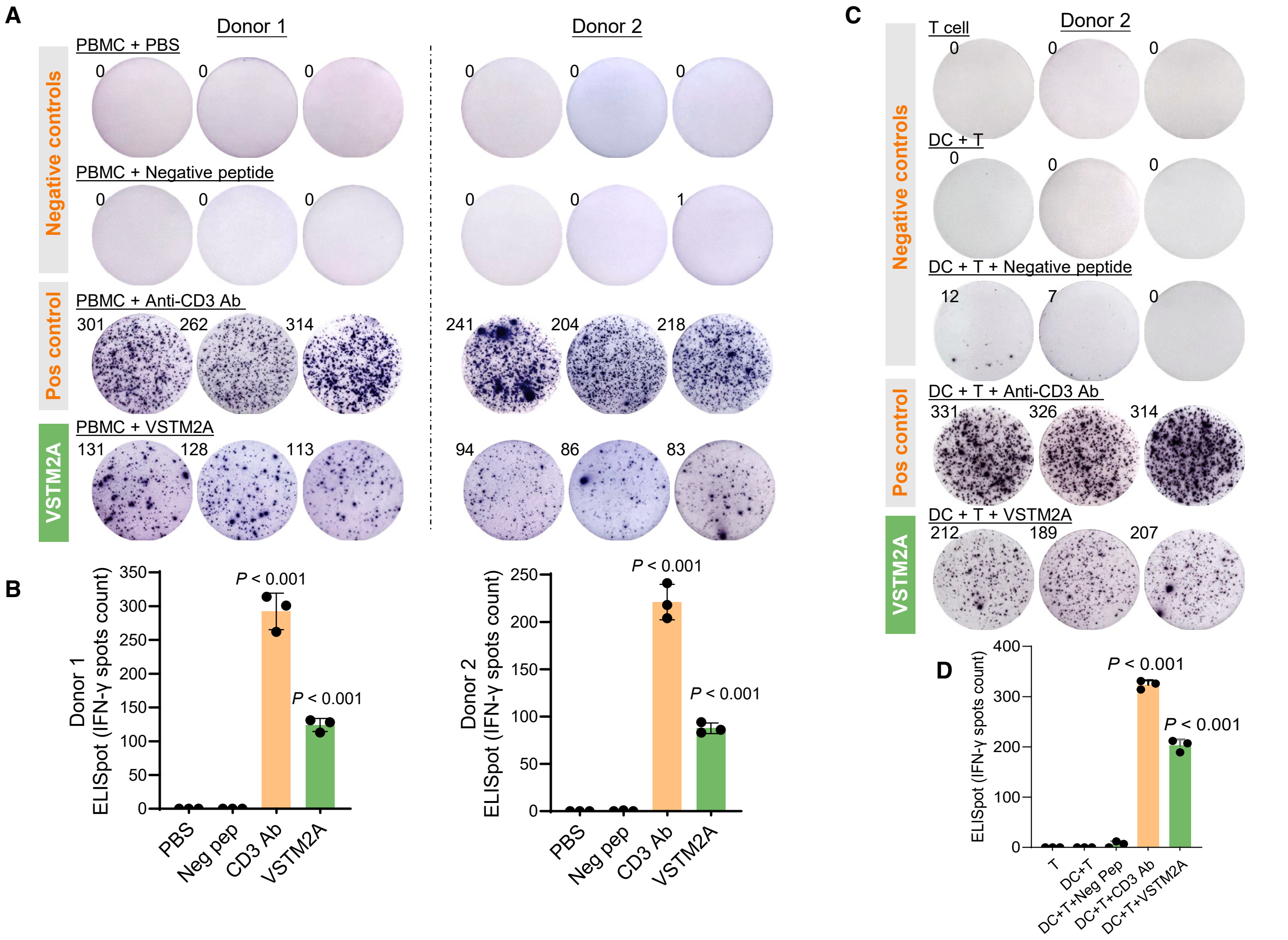

Figure 6. VSTM2A promotes IFN-γ secretion by PBMCs

(A) Images of the ELISpot assay showing IFN-γ secretion by PBMCs following 24 h stimulation with the anti-CD3 antibody or VSTM2A recombinant protein (1 μg/mL). (B) Summary of the IFN-γ spot number generated in (A). (C) Images of the ELISpot assay showing the IFN-γ secretion by DC:CD8$^+$ T cell co-culture following 24 h stimulation with the anti-CD3 antibody or VSTM2A. Autologous DCs and CD8$^+$ T cells were used in the analysis. (D) Summary of the IFN-γ spot number generated in (C). Statistical analysis was performed against Actin peptide using an unpaired t test. Data are presented as mean values ± SD.

quantitative PCR (qPCR) and western blot analysis (Figures 5E and S3A). Using the recombinant VSTM2A protein as a standard curve, we quantified the VSTM2A protein in each specimen based on its densitometry data. Considering that the average physical density of human gastrointestinal tract tissue is 1.03 g/mL,[15] we calculated the physiological level of VSTM2A in adjacent normal colon ranges of 0.93–4.0 μg/mL (Figure 5E). In keeping with our previous report, CRC tissues and cultured two-dimensional (2D) cell lines seldom express VSTM2A (Figures S3B, S4A, and S4B). We therefore chose 1 μg/mL recombinant human VSTM2A (carrier free) for the PD-L1 signaling study. To investigate if Wnt signaling is affected under such experimental conditions, we treated T cells isolated from peripheral blood mononuclear cells (PBMCs) of a healthy donor with VSTM2A protein up to 4 h and detected the level of LRP6 phosphorylation. A high concentration of VSTM2A (10 μg/mL) significantly suppresses LRP6 phosphorylation (Ser1490) in T cells at both time points (30 min and 4 h), whereas a low concentration of VSTM2A (1 μg/mL) shows no effect on LRP6 phosphorylation in T cells, suggesting that 1 μg/mL VSTM2A will not interfere with Wnt signaling (Figure 5F). In addition, 1 μg/mL VSTM2A had a limited effect on T cell apoptosis (Figure S5). We performed an *in vitro* direct tumor cell killing assay by co-culturing CRC cells with T cells. Target cells HCT116 with endogenous PD-L1 expressing was transfected with GFP-2A-luciferase plasmid and incubated with effector cells (wild-type T cells from the healthy donor) at a ratio (E/T ratio) ranging from 4:1 to 32:1 for 24 h, followed by a measurement of cell viability. Compared with phosphate-buffered saline (PBS) control, 1 μg/mL VSTM2A significantly elevated the lytic ability of wild-type T cells against HCT116 cells (Figures 5G and S6). We detected the level of cytolytic protein granzyme B and effector cytokine interferon

(IFN)-γ in the co-culture supernatant using ELISA. Increased secretion of granzyme B and IFN-γ was observed in the presence of 1 μg/mL VSTM2A, suggesting that VSTM2A promoted the cytotoxicity of T cells (Figures 5H and 5I).

To further investigate the immune-activation functions of VSTM2A in a clinically relevant model, we quantified the cytokine secretion by human PBMCs using ELISpot assay. To eliminate any false positive immune response caused by potential contaminations during peptide synthesis, we synthesized a peptide derived from human Actin protein as a negative control in addition to the PBS control. ELISpot analysis was performed using PBMCs isolated from three donors, with each group running in triplicate wells. Indeed, our result showed that VSTM2A recombinant protein triggered significant IFN-γ release at a concentration of 1 μg/mL compared with negative controls in both PBMCs ($p < 0.0001$, Figures 6A and 6B). To further decipher the source of IFN-γ secretion, we isolated pan-dendritic cells (DCs) and co-cultured them with autologous CD8^{+} T cells for 24 h. Consistently, treatment with VSTM2A (1 μg/mL) significantly promoted INF-γ secretion in comparison with negative controls ($p < 0.0001$, Figures 6C and 6D), indicating that cytotoxic T lymphocytes may release IFN-γ in response to VSTM2A. In addition, we used durvalumab (anti-PD-L1 ICI) to neutralize the PD1/PD-L1 interaction and evaluate the effect of VSTM2A in human PBMCs. 1×10^{5} freshly isolated PBMCs from a healthy donor were incubated with VSTM2A (1 μg/mL) in the presence of 10 μg/mL durvalumab or an IgG isotype control for 48 h in each ELISpot well. VSTM2A significantly increased the IFN-γ spot numbers compared with the IgG treatment. This effect of VSTM2A was abolished when cells were co-treated with durvalumab (Figure S7), suggesting that VSTM2A could induce a pro-immunogenic effect that requires the interaction with PD-L1. In summary, we identified the tumor-extrinsic role of VSTM2A in regulating T cell activation through antagonizing PD-1/PD-L1-mediated suppressive signaling. Our study creates foundations for facilitating the use of VSTM2A protein for immunotherapeutic interventions against CRC.

DISCUSSION

Cancer immunotherapy against immune checkpoint pathway PD-1 and its ligand PD-L1 have revolutionized and achieved unprecedented success in the past few decades. However, systemic administration of ICI mAb is associated with significant toxicities due to enhanced activation of autoreactive T cells, relatively low solid tumor penetration rates, significant clearance by tumor-associated macrophages, and patients' innate or developed resistance during treatment.[7] In this study, we deciphered a hitherto unrecognized role of VSTM2A as a small innate antagonist that sterically abrogates the PD-L1/PD-1 interaction, leading to the alleviation of immunosuppression on cytotoxic T cells. We provided a series of *in vivo*, *in vitro*, and *ex vivo* evidence to support this notion. First, we observed the positive correlation between VSTM2A and CD8a^{+} using a *Vstm2a*$^{+/-}$ KO mice model, C57BL/6 synergistic mice xenografts, and clinical CRC specimens. Bioinformatics further indicated a connection between VSTM2A mRNA and immune infiltration scores in both physiological and pathological conditions. Next, supported by LC-MS/MS, we identified PD-L1 as a membrane receptor of VSTM2A. The VSTM2A/PD-L1 interaction was validated by a series of *in vitro* biochemistry assays, including coIP, domain mapping, and titration ELISA. We determined the binding pattern and kinetics between VSTM2A and PD-L1 proteins from their IgV domains. The published K_D values for human PD-1/PD-L1 binding measured using titration ELISA ranges from 10 to 50 nM (technical notes from Cisbio and ACROBiosystems), whereas the apparent K_D of VSTM2A binding with PD-L1 ranges from 0.7 to 2.5 nM, suggesting that the binding affinity of VSTM2A/PD-L1 is higher. The biological consequence of VSTM2A/PD-L1 was subsequently demonstrated using two *in vitro* APC-T cell co-culture models. Finally, tumor killing assay and *ex vivo* data from the human PBMC studies validated the immunoenhancing effects modulated by VSTM2A. Efficient anti-tumor immune responses require robust and efficient induction of cytotoxic CD8a^{+} T cells. We observed that VSTM2A significantly enhances immune activation by releasing significant granzyme B and IFN-γ cytokines, supporting VSTM2A as a promising agent in the fight against CRC by interrupting the PD-L1/PD-1 axis.

Our study provided potential immunotherapeutic options for CRC treatment. First of all, the predicted molecular mass of VSTM2A (∼26.5 kDa) is only 1/6 of a mAb (150–160 kDa). Previous studies of nanobodies (∼15 kDa) suggested that the small molecular weight conferred the nanobody with strong tissue penetration[16]; it is possible that VSTM2A could have better penetration in solid tissues compared with mAbs based on size. More importantly, our studies reveal the multi-function of VSTM2A in tumor-intrinsic Wnt/β-catenin pathway inhibition and tumor-extrinsic immune activation via antagonizing PD-L1 signaling. Compared with patients with dMMR/MSI-H-type CRC featuring increased levels of tumor-associated antigens and tumor-infiltrating lymphocytes (immune-hot tumor), approximately 85% of patients with CRC (proficient in MMR) are lack significant T cell infiltration in the tumor (cold tumor) and may not benefit from current ICI treatments.[17,18] Our study demonstrated that VSTM2A positively correlated with CD8^{+} T cell infiltration and antagonized the PD-L1/PD-1 inhibitory signal; therefore, administration of VSTM2A would inhibit CRC tumor cell growth directly while favoring lymphocyte tumor infiltration and activation. Next, the structure of the VSTM2A/PD-L1 interaction may provide inspiration for the design of new single-domain antibodies (sdAbs) against PD-L1. sdAb is the single variable domain fragment of heavy chain that only binds to the epitope via complementarity-determining regions (CDRs).[19] Recent study has developed a panel of 16 novel anti-PD-L1 sdAbs that could complete the inhibition of PD-L1 binding to PD-1 at nanomole to micromole affinities.[20] The structural feature of VSTM2A and its affinity with PD-L1 at the picomole to nanomole level may offer a potential avenue to design more selective and stronger sdAbs against PD-L1. Finally, previous studies suggested that PD-1/PD-L1 axis blockade might be a potential approach to optimize chimeric antigen receptor (CAR) T cell metabolism and hence expand their cytotoxicity.[21] PD-L1 blockade could also enhance the anti-tumor efficacy of NK cells.[22] Future studies are needed to test

whether the VSTM2A protein could also enhance the potency of autologous tumor-reactive T cell therapy and NK cell-based cancer immunotherapy. This research will lead to the engineering of a new generation of CAR molecules with VSTM2A as a transgenic "payload" protein to enhance the therapy potency.

Similar to other bio-active compounds, such as transforming growth factor β1 (TGF-β1) and chloroquine,[23] there is a certain biphasic dose effect (dose-related cytotoxicity) in using VSTM2A that should not be neglected. We demonstrated that human PBMCs could be activated by 1 μg/mL VSTM2A protein and release significant amounts of IFN-γ (Figure 5). However, flow cytometry revealed that higher concentrations of VSTM2A protein (>13.3 μg/mL, equal to 518.7 nM) for 24 h significantly triggered PBMC apoptosis in a dose-dependent manner (Figure S5). Moreover, we observed that less than 1 μg/mL VSTM2A recombinant protein significant promoted the NFAT-luciferase activity in a PD-1/PD-L1 blockade bioassay at 24 h, whereas higher concentrations of VSTM2A (>4 μg/mL, equal to 156 nM) started to induce signal inhibition (data not shown). Consistently, a similar observation was mentioned by the recombinant VSTM2A manufacturer in a non-peer-reviewed preliminary test using ELISA detection of IFN-γ secretion as the endpoint measurement (dose >10 μg/mL). Therefore, we speculate that the dose-response curve of the VSTM2A protein is a nonlinear inverted U shape: a low dose of VSTM2A activates immune cell IFN-γ secretion, whereas a high VSTM2A concentration shows toxicity effects against PBMCs. Whether this hormesis effect involves solely the VSTM2A/PD-L1 interaction or other receptors/pathways is still unknown. Given the observed inhibition concentration of VSTM2A against the PD-L1/PD-1 complex is relatively low, it is possible to optimize the dosing of VSTM2A at picomolar concentrations to achieve the desired immunoenhancing effects on PBMCs while avoiding significant adverse effects *in vivo*. To facilitate the use of the VSTM2A protein in the clinic, future pharmacologic studies are needed to comprehensively investigate the safety and tolerability of administering the VSTM2A protein using animal models prior to first-in-human trials.

PD-L1 can elicit diverse functions beyond immunosuppressive signaling pathways in cancer, implying that the VSTM2A/PD-L1 interaction could have broad functional significance in other conditions. Previous study revealed that VSTM2A mainly distributed in the gastrointestinal tract as well as in the central nervous system in adult humans.[24] Animal research indicated that remarkably high *Vstm2a* expression in mouse C57BL/6J brain and spinal cord started at embryonic day (E)14 and maintained a relatively high level throughout adulthood.[25] The coincidence between strong *Vstm2a* expression and active neurogenesis during mice early brain development provides a potential explanation for the unavailability of homozygous *Vstm2a* KO mice in our animal breeding. Recent study suggested that reduced PD-L1/PD-1 signaling could promote hippocampal neuronal excitability and memory behaviors in an adult mouse model.[26] In addition, previous studies highlighted the role of VSTM2A in amplifying adipogenic commitment by acting upon the PI3K/mTOR- and cAMP-dependent signaling pathways.[27,28] Given the PD-L1/PD-1, mTOR, and Wnt pathways play essential roles in the proper differentiation of cortical layers, cognitive development, brain injury, and Alzheimer's disease, it is plausible that VSTM2A is indispensable in the maintenance of human central nervous system. Nonetheless, the role of VSTM2A/PD-L1's interaction in vertebrates' neurogenesis requires further investigation.

Our research has several limitations. Despite VSTM2A being able to interact with both PD-1 ligands (PD-L1 and PD-L2), the binding affinity between VSTM2A and PD-L2 was not determined in the current study. Since the two PD-1 ligands exhibit different expression pattens and are varied in function,[29] the biological consequence of VSTM2A in competition with PD-L2/PD-1 signaling requires further investigation. Moreover, we observed that recombinant VSTM2A protein induced a pro-immunogenic effect in human PBMCs, yet the mechanism of VSTM2A in triggering such an effect is still unknown. Last but not least, our data demonstrated the potential of VSTM2A in immune regulation in KO mouse and human PBMC models; however, due to a lack of clinical specimens, we were unable to evaluate the VSTM2A expression and efficacy and prognosis of existing ICI-related therapies in patients with CRC. The translational values of VSTM2A (as a biomarker, a biological drug, and CAR-T therapy) require further prospective analysis.

In summary, our study demonstrated that VSTM2A sterically blocks PD-1 binding to PD-L1 at a picomole to nanomole affinity, which leads to the enhanced anti-tumor effect of cytotoxic T cells. Our concrete evidence establishing VSTM2A as a CRC immunotherapeutic agent holds the promise for better therapeutic options for patients with advanced CRC.

MATERIALS AND METHODS

Human CRC samples

Our tissue microarray cohort consists of 208 patients with histologically confirmed CRC who underwent surgery at the Prince of Wales Hospital, Hong Kong. The patient characteristics are summarized in Table S1. Two publicly available cohorts (TCGA-COAD/READ and GEO: GSE17538) containing 557 and 585 patients with CRC, respectively, were used for validation. RNA-seq data of normal colon samples from TCGA cohort and the GTEx datasets were used in correlation analysis.

Human and mouse cell culture

RKO (ATCC: CRL-2577) and 293TN (System Biosciences: LV900A-1) lines were maintained in Dulbecco's modified Eagle medium (DMEM) (Gibco, Thermo Fisher Scientific) supplemented with 10% fetal bovine serum (FBS) and 100 U/mL penicillin-streptomycin (Thermo Fisher Scientific). BALB/c mouse spleen cells and MC38 and DO11.10 T cells (a gift from Prof. Tasuku Honjo, Kyoto University) were cultured in RPMI-1640 Medium (Thermo Fisher Scientific) supplemented with 10% FBS and 100 U/mL penicillin-streptomycin. Cells were cultured in a 37°C, 5% CO_2 incubator.

VSTM2A conditioned media and recombinant protein used in this study

VSTM2A conditioned medium and control medium were harvested from 293TN cells transiently transfected with VSTM2A expression or empty control constructs. Briefly, 293TN cells were seeded in 10 cm dishes and transfected with 16 μg of p3.1-VSTM2A or empty vector using Lipofectamine 2000 as directed by the manufacturer (Thermo Fisher Scientific). Conditioned medium was harvested 48 h after transfection. Recombinant human VSTM2A protein (10037-VT-050, carrier-free version) was purchased from R&D Systems.

xCell and CIBERSORTx *in silico* analysis

The ImmuneScores of the CRC cases were determined by the xCell package.[12] 244 cases from TCGA-COAD dataset were used to estimate the relative proportions of 22 types of infiltrating immune cells by the CIBERSORTx algorithm (https://cibersortx.stanford.edu/).[30] Hierarchical clustering of correlations among the 22 immune cell compositions with high vs. low VSTM2A expression was performed and plotted.

Vstm2a KO mice model

Whole-body heterozygous *Vstm2a*$^{+/-}$ C57BL/6 mice were generated by the KO of exon 2 using Cre/*LoxP*-based technology (Biocytogen). Genotyping was performed on each mouse before grouping. Male *Vstm2a*$^{+/-}$ (n = 14) and male wild-type littermate mice (n = 18) were employed for the AOM/DSS model. To induce CRC, mice were injected once with AOM (10 mg/kg, intraperitoneally) at 6 weeks of age. One week after AOM injection, three cycles of DSS treatment (1.5% in drinking water for 5 days) were given to mice, with a 1 week break between each cycle. Mice were sacrificed 80 days after the first AOM injection. Mouse weight was measured before tissue harvesting. Visible nodules on the colon were measured using a caliber. Colon tissues were immediately fixed in 10% formalin. Histology was scored after hematoxylin and eosin (H&E) staining by a pathologist blinded to the nature of samples.

Syngeneic mouse xenograft model

MC38 cells expressing empty vector or the mouse *Vstm2a* gene (5×10^6 cells in 0.1 mL PBS) were injected subcutaneously into the dorsal flank of 5-week-old female C57BL/6 mice. Subcutaneous tumor size was measured with a digital caliper. Mice were sacrificed for xenograft examination at 4 weeks post-tumor inoculation.

PD-1/PD-L1 blockade bioassay

The PD-1/PD-L1 Blockade Bioassay (Promega) was performed according to the manufacturer's instructions. In brief, PD-1 effector cells expressing human PD-1 and a luciferase reporter driven by an NFAT-RE was co-cultured with PD-L1 aAPC/CHO-K1 with an engineered cell surface protein that could activate cognate T cell receptors (TCRs) in an antigen-independent manner. The bioluminescent signal was quantified using the Bio-Glo Luciferase Assay System (Promega) in the presence of recombinant human VSTM2A (R&D Systems, 10037-VT) according to the manufacturer's instructions.

Titration ELISA

The dissociation constant between VSTM2A and PD-L1 was determined by titration ELISA as described previously.[8] In brief, 5 μg/mL recombinant human PD-L1 chimera protein (R&D Systems, 156-B7) was immobilized on an ELISA plate (50 μL per well) at 4°C for 12 h. The coated plate was washed with PBS and blocked by 5% bovine serum albumin (BSA) solution in PBS for 12 h at 4°C. Recombinant human VSTM2A protein (R&D Systems, 10037-VT) serial dilution (a start concentration of 100 μg/mL) was incubated in an ELISA plate in duplicate for 2 h at room temperature. After removing non-bound VSTM2A with PBS, the ELISA plate was fixed by 3,3′-dithiobis(sulfosuccinimidyl propionate) (DTSSP). PD-L1-bound VSTM2A was determined by ELISA with Alkaline Phosphatase Yellow (pNPP) Liquid Substrate (Sigma-Aldrich) at OD_{405}. Apparent K_D values were calculated by fitting specific signals to a one-site specific binding curve using GraphPad Software. The competition assay was tested using the PD-1 [Biotinylated]: PD-L1 Inhibitor Screening ELISA Assay Pair (Acro Biosystems, EP-101).

IHC and western blot

IHC staining of VSTM2A and CD8a was performed using the EnVision system (Agilent). The results were scored by a pathologist. Proteins were separated on 10% SDS-PAGE and transferred onto polyvinylidene difluoride membranes (Sigma-Aldrich). Blots were immunostained with primary antibody and secondary antibody. Antibodies used in this study are listed in Table S2.

qPCR

qPCR was performed with the Power SYBR Green PCR Master Mix (Thermo Fisher Scientific) and QuantStudio 7 Flex Real-Time PCR System (Thermo Fisher Scientific). Primer sequences are listed in Table S2. Target gene expression was normalized to GAPDH or β-actin expression and quantified using the delta-Ct method.

coIP and MS analysis

RKO cells were seeded on 100 mm polyethylenimine (PEI, Sigma-Aldrich)-coated dishes. After 4 h incubation with DMEM serum-free medium at 37°C, cells were chilled on ice, followed by treatment with His-tag VSTM2A conditioned medium for 1 h at 4°C. The cells were then incubated with 1.6 mM cell-impermeable crosslinker DTSSP (Sigma-Aldrich) for 2 h on ice. Whole-cell lysates were incubated with 400 μL Ni-NTA agarose beads (Qiagen) for 12 h. Potential binding proteins were eluted with 1.5 mL RIPA buffer containing 250 mM imidazole and precipitated with 10% trichloroacetic acid. The sample was loaded onto 10% SDS-PAGE for separation followed by Coomassie blue staining. The gel was excised into 11 segments and submitted for shotgun proteomics analyses using an EASY-nL CTM 1200 UHPLC system (Thermo Fisher Scientific) coupled with an Orbitrap Q Exactive HF-X mass spectrometer (Thermo Fisher Scientific) operating in the data-dependent acquisition mode. The resulting spectra from each fraction were searched separately against the Homo_sapiens_uniprot (169,389 sequences) database by the search engine Proteome Discoverer 2.2 (PD 2.2, Thermo Fisher Scientific).

Annexin V apoptosis analysis

Human PBMCs were treated with various doses of recombinant human VSTM2A proteins and cultured for 24 h at 37°C with 5% CO_2. Untreated PBMCs was used as a negative control. Cell viability was determined using the BD Pharmingen FITC Annexin V Apoptosis Detection Kit I on the BD LSRFortessa cell analyzer with acquisition criteria of 10,000 events for each tube. Data were analyzed using FlowJo software (Treestar).

Immunotyping

Tissue was dissected using 1% collagenase D (Sigma-Aldrich) in the PBS supplied with 1% BSA and 0.005% DNAse I (Sigma-Aldrich) at 37°C for 30 min. The digested tissue was gently ground with a 10 mL syringe plunger on the 70 μm Cell-Strainer (BD Biosciences, San Jose, CA). The suspension was centrifuged at 700*g*, 4°C for 10 min. Cell pallets were resuspended using 1% BSA for 30 min and stained for extracellular markers. The fluorescent antibodies used in this study are shown in Table S2. Cells were washed with PBS and analyzed on a BD LSRFortessa cell analyzer. Data were analyzed using FlowJo software (Treestar).

Labeling of HCT116 cells with GFP-luciferase

Lentivirus particles were produced in 293T cells using polyethyleneimine (PEI; Sigma-Aldrich) transfection. A total of 24 μg plasmids comprising pGL lentiviral plasmid and two packaging plasmids, psPAX2 and pMD.2G, were co-transduced into 293TN cells in a 10 cm dish with 72 μg PEI. Lentivirus-containing supernatants were harvested at 48 h post-transfection. Supernatant was collected through centrifuge at 2,000 rpm for 10 min and incubated with HCT116 cells for 48 h. Labeling efficiency was determined using flow cytometer.

Isolation and expansion of primary human T lymphocytes

PBMCs were separated via density gradient centrifugation (Lymphoprep, StemCell Technologies). Primary human T cells were enriched from PBMCs via negative selection using the Pan T Isolation Kit (Miltenyi Biotec). Freshly isolated T cells were stimulated using T Cell TransAct (Miltenyi Biotec). Approximately 24 h after activation, T cells were cultured with GT-T551 H3 medium supplemented with human IL-2 (300 IU/mL) and 5% FBS.

In vitro tumor killing assays

The target cells HCT116-GL (10^4 per well) were incubated with wild-type T cells in complete RPMI-1640 medium without IL-2 at the indicated ratios in triplicate wells in 96-well white flat-bottom plates. 1 μg/mL VSTM2A or PBS control was added into each well. Target cell viability was monitored 24 h later by adding the substrate Luciferin (Cayman Chemical). The viability percentage (%) was equal to the experimental signal/maximal signal, and the killing percentage was equal to 100 − viability percentage.

Stimulation of DO11.10 T hybridoma cells

DO11.10 T hybridoma cells (5×10^4 cells/well) were stimulated with APCs (1×10^4 cells/well) pulsed with $OVA_{323-339}$ peptide (Sigma-Aldrich) in 96-well round-bottom plate as described before.[31] The concentration of IL-2 in the culture supernatant was determined by ELISA (BioLegend).

IFN-γ ELISpot assay

Human PBMCs, autologous DCs, and $CD8^+$ T cells were used in the ELISpot assay. PBMCs were freshly isolated from human buffy coats by centrifugation over Ficoll-Paque PLUS (Cytiva, 17144002). DCs were isolated using the Pan-DC Enrichment kit (Miltenyi Biotec, 130-100-777). $CD8^+$ T cells were enriched using the $CD8^+$ T cell Isolation kit (Miltenyi Biotec, 130-096-495). Human IFN-γ was evaluated using the ELISpot Plus Human IFN-γ (ALP) kit (Mabtech). Anti-human CD3 mAb (Mabtech) and human Actin within the aa region 1–9 (MCDEDETTA, purity >98%, trifluoroacetic acid residue <1%, and endotoxin level ≤0.01 EU/μg) were used as positive and negative controls, respectively. Spot development was performed according to the manufacturer's instructions and counted using ImageJ software.

Statistics

Statistical analysis was performed in GraphPad Prism (v.10). Categorical data were compared with the chi-squared test. Continuous data were compared with Student's t test or Mann-Whitney U test when appropriate. p values < 0.05 were considered statistically significant.

Study approval

The human sample collection protocol was approved by The Joint Chinese University of Hong Kong - Hospital Authority New Territories East Cluster Clinical Research Ethics Committee. All animal experimentation at the Chinese University of Hong Kong complies with the University Animal Experimentation Ethics Committee.

DATA AND CODE AVAILABILITY

All data associated with this study are present in the paper.

ACKNOWLEDGMENTS

This study was supported by RGC-CRF Hong Kong (C4039-19GF and C4008-23W). This study was supported in part by the Hong Kong Research Grants Council-General Research Fund (14115123) and CUHK Direct Grant (4054798).

AUTHOR CONTRIBUTIONS

Y.D. and J.J.L. performed the experiments and drafted the manuscript; Y.Z. performed KO mice model-based experiments; W.K. and A.H.K.C. performed the histological evaluation as pathologists; S.L. and Y.H. performed experiments; R.L. and C.C.W. revised the manuscript; N.W., S.S.M.N., and J.Y. supervised the study; and J.Y. and Y.D. secured the research grants.

DECLARATION OF INTERESTS

The authors declare no competing interests.

SUPPLEMENTAL INFORMATION

Supplemental information can be found online at https://doi.org/10.1016/j.ymthe.2024.09.023.

REFERENCES

1. Siegel, R.L., Giaquinto, A.N., and Jemal, A. (2024). Cancer statistics, 2024. CA. Cancer J. Clin. *74*, 12–49.

2. Cervantes, A., Adam, R., Roselló, S., Arnold, D., Normanno, N., Taïeb, J., Seligmann, J., De Baere, T., Osterlund, P., Yoshino, T., et al. (2023). Metastatic colorectal cancer: ESMO Clinical Practice Guideline for diagnosis, treatment and follow-up. Ann. Oncol. *34*, 10–32.

3. Le, D.T., Durham, J.N., Smith, K.N., Wang, H., Bartlett, B.R., Aulakh, L.K., Lu, S., Kemberling, H., Wilt, C., Luber, B.S., et al. (2017). Mismatch repair deficiency predicts response of solid tumors to PD-1 blockade. Science *357*, 409–413.

4. Le, D.T., Uram, J.N., Wang, H., Bartlett, B.R., Kemberling, H., Eyring, A.D., Skora, A.D., Luber, B.S., Azad, N.S., Laheru, D., et al. (2015). PD-1 Blockade in Tumors with Mismatch-Repair Deficiency. N. Engl. J. Med. *372*, 2509–2520.

5. Overman, M.J., McDermott, R., Leach, J.L., Lonardi, S., Lenz, H.-J., Morse, M.A., Desai, J., Hill, A., Axelson, M., Moss, R.A., et al. (2017). Nivolumab in patients with metastatic DNA mismatch repair-deficient or microsatellite instability-high colorectal cancer (CheckMate 142): an open-label, multicentre, phase 2 study. Lancet Oncol. *18*, 1182–1191.

6. Overman, M.J., Lonardi, S., Wong, K.Y.M., Lenz, H.-J., Gelsomino, F., Aglietta, M., Morse, M.A., Van Cutsem, E., McDermott, R., Hill, A., et al. (2018). Durable Clinical Benefit With Nivolumab Plus Ipilimumab in DNA Mismatch Repair–Deficient/Microsatellite Instability–High Metastatic Colorectal Cancer. J. Clin. Oncol. *36*, 773–779.

7. McGowan, E., Lin, Q., Ma, G., Yin, H., Chen, S., and Lin, Y. (2020). PD-1 disrupted CAR-T cells in the treatment of solid tumors: Promises and challenges. Biomed. Pharmacother. *121*, 109625.

8. Dong, Y., Zhang, Y., Kang, W., Wang, G., Chen, H., Higashimori, A., Nakatsu, G., Go, M., Tong, J.H., Zheng, S., et al. (2019). VSTM2A suppresses colorectal cancer and antagonizes Wnt signaling receptor LRP6. Theranostics *9*, 6517–6531.

9. Kundapura, S.V., and Ramagopal, U.A. (2019). The CC′ loop of IgV domains of the immune checkpoint receptors, plays a key role in receptor:ligand affinity modulation. Sci. Rep. *9*, 19191.

10. Manieri, N.A., Chiang, E.Y., and Grogan, J.L. (2017). TIGIT: A Key Inhibitor of the Cancer Immunity Cycle. Trends Immunol. *38*, 20–28.

11. Guo, X., Zhang, Y., Wang, P., Li, T., Fu, W., Mo, X., Shi, T., Zhang, Z., Chen, Y., Ma, D., and Han, W. (2012). VSTM1-v2, a novel soluble glycoprotein, promotes the differentiation and activation of Th17 cells. Cell. Immunol. *278*, 136–142.

12. Aran, D., Hu, Z., and Butte, A.J. (2017). xCell: digitally portraying the tissue cellular heterogeneity landscape. Genome Biol. *18*, 220.

13. Pascolutti, R., Sun, X., Kao, J., Maute, R.L., Ring, A.M., Bowman, G.R., and Kruse, A.C. (2016). Structure and Dynamics of PD-L1 and an Ultra-High-Affinity PD-1 Receptor Mutant. Structure *24*, 1719–1728.

14. Zak, K.M., Grudnik, P., Magiera, K., Dömling, A., Dubin, G., and Holak, T.A. (2017). Structural Biology of the Immune Checkpoint Receptor PD-1 and Its Ligands PD-L1/PD-L2. Structure *25*, 1163–1174.

15. Noblet, C., Delpon, G., Supiot, S., Potiron, V., Paris, F., and Chiavassa, S. (2018). A new tissue segmentation method to calculate 3D dose in small animal radiation therapy. Radiat. Oncol. *13*, 32.

16. Yang, X.M., Lin, X.D., Shi, W., Xie, S.X., Huang, X.N., Yin, S.H., Jiang, X.B., Hammock, B.D., Xu, Z.P., and Lu, X.L. (2023). Nanobody-based bispecific T-cell engager (Nb-BiTE): a new platform for enhanced T-cell immunotherapy. Signal Transduct. Targeted Ther. *8*, 328.

17. Galon, J., and Bruni, D. (2019). Approaches to treat immune hot, altered and cold tumours with combination immunotherapies. Nat. Rev. Drug Discov. *18*, 197–218.

18. Lizardo, D.Y., Kuang, C., Hao, S., Yu, J., Huang, Y., and Zhang, L. (2020). Immunotherapy efficacy on mismatch repair-deficient colorectal cancer: From bench to bedside. Biochim. Biophys. Acta Rev. Cancer *1874*, 188447.

19. Tang, H., Gao, Y., and Han, J. (2023). Application Progress of the Single Domain Antibody in Medicine. Int. J. Mol. Sci. *24*, 4176.

20. Kang-Pettinger, T., Walker, K., Brown, R., Cowan, R., Wright, H., Baravalle, R., Waters, L.C., Muskett, F.W., Bowler, M.W., Sawmynaden, K., et al. (2023). Identification, binding, and structural characterization of single domain anti-PD-L1 antibodies inhibitory of immune regulatory proteins PD-1 and CD80. J. Biol. Chem. *299*, 102769.

21. Daei Sorkhabi, A., Mohamed Khosroshahi, L., Sarkesh, A., Mardi, A., Aghebati-Maleki, A., Aghebati-Maleki, L., and Baradaran, B. (2023). The current landscape of CAR T-cell therapy for solid tumors: Mechanisms, research progress, challenges, and counterstrategies. Front. Immunol. *14*, 1113882.

22. Bai, R., and Cui, J. (2022). Burgeoning Exploration of the Role of Natural Killer Cells in Anti-PD-1/PD-L1 Therapy. Front. Immunol. *13*, 886931.

23. Calabrese, E.J. (2013). Biphasic dose responses in biology, toxicology and medicine: Accounting for their generalizability and quantitative features. Environ. Pollut. *182*, 452–460.

24. Lonsdale, J., Thomas, J., Salvatore, M., Phillips, R., Lo, E., Shad, S., Hasz, R., Walters, G., Garcia, F., Young, N., et al. (2013). The Genotype-Tissue Expression (GTEx) project. Nat. Genet. *45*, 580–585.

25. Visel, A., Thaller, C., and Eichele, G. (2004). GenePaint.org: an atlas of gene expression patterns in the mouse embryo. Nucleic Acids Res. *32*, 552–556.

26. Zhao, J., Bang, S., Furutani, K., McGinnis, A., Jiang, C., Roberts, A., Donnelly, C.R., He, Q., James, M.L., Berger, M., et al. (2023). PD-L1/PD-1 checkpoint pathway regulates hippocampal neuronal excitability and learning and memory behavior. Neuron *111*, 2709–2726.e9.

27. Secco, B., Camiré, É., Brière, M.-A., Caron, A., Billong, A., Gélinas, Y., Lemay, A.-M., Tharp, K.M., Lee, P.L., Gobeil, S., et al. (2017). Amplification of Adipogenic Commitment by VSTM2A. Cell Rep. *18*, 93–106.

28. Al Dow, M., Silveira, M.A.D., Poliquin, A., Tribouillard, L., Fournier, É., Trébaol, E., Secco, B., Villot, R., Tremblay, F., Bilodeau, S., and Laplante, M. (2021). Control of adipogenic commitment by a STAT3-VSTM2A axis. Am. J. Physiol. Endocrinol. Metab. *320*, E259–E269.

29. Bardhan, K., Anagnostou, T., and Boussiotis, V.A. (2016). The PD1:PD-L1/2 Pathway from Discovery to Clinical Implementation. Front. Immunol. *7*, 550.

30. Cancer Genome Atlas Network (2012). Comprehensive molecular characterization of human colon and rectal cancer. Nature *487*, 330–337.

31. Sugiura, D., Maruhashi, T., Okazaki, I.M., Shimizu, K., Maeda, T.K., Takemoto, T., and Okazaki, T. (2019). Restriction of PD-1 function by cis-PD-L1/CD80 interactions is required for optimal T cell responses. Science *364*, 558–566.

YMTHE, Volume *32*

Supplemental Information

VSTM2A reverses immunosuppression in colorectal cancer by antagonizing the PD-L1/PD-1 interaction

Yujuan Dong, Jiaxun Jade Liu, Yunfei Zhou, Wei Kang, Shanglin Li, Alvin H.K. Cheung, Yi Hu, Rui Liao, Nathalie Wong, Chi Chun Wong, Simon S.M. Ng, and Jun Yu

Figure S1

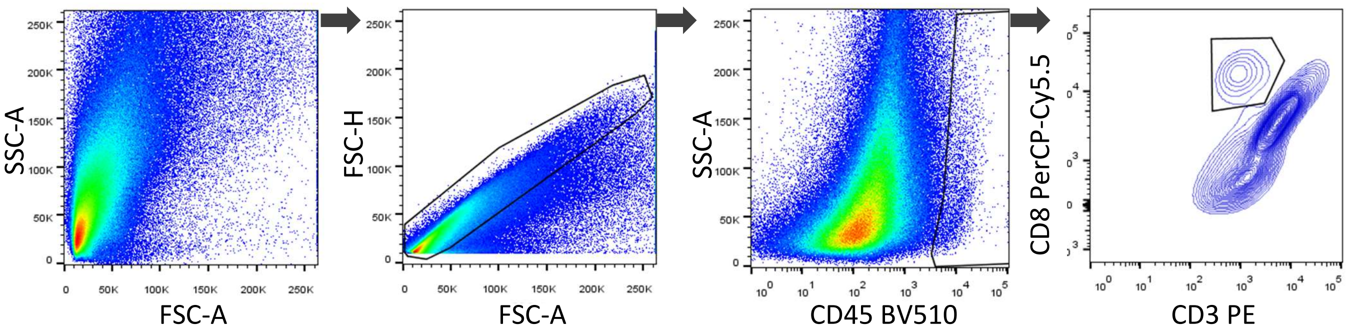

Figure S1. Gating strategy of immune cell subsets in AOM/DSS immunocompetent mice model.

Figure S2

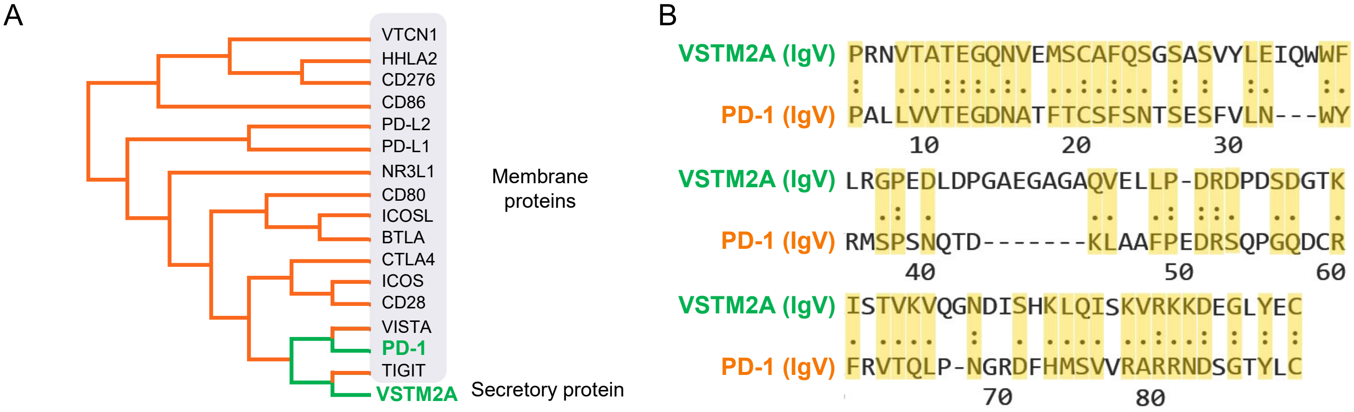

Figure S2. (A) Phylogenetic tree based on the IgV domain sequences of B7 and CD28 protein families. **(B)** Protein sequence alignments of PD-1 and VSTM2A IgV domain.

Figure S3

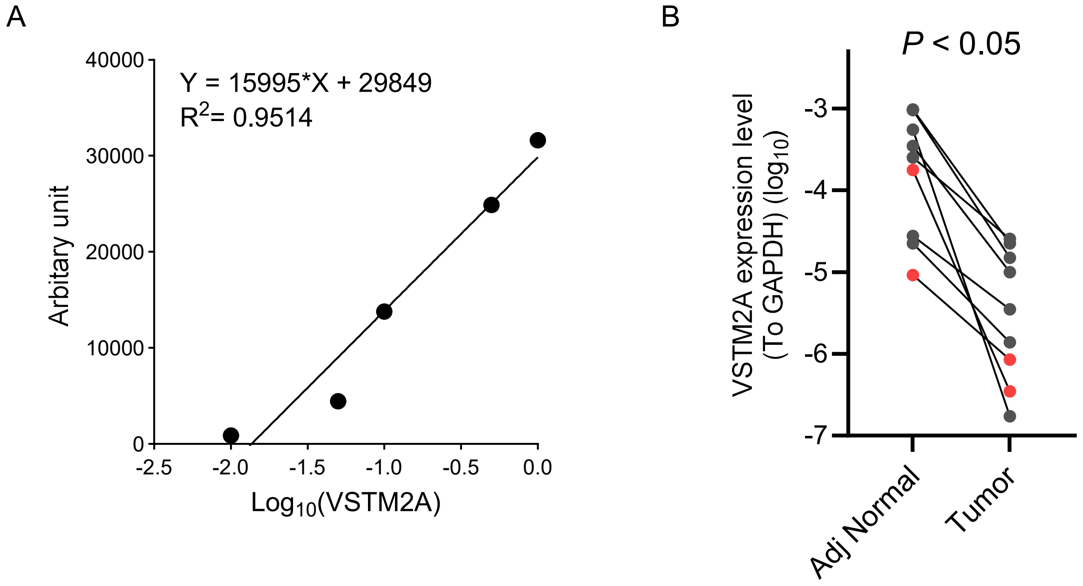

Figure S3. (A) Protein standard curve using Recombinant human VSTM2A. **(B)** VSTM2A mRNA in primary tumour and matched adjacent nontumour tissue from CRC patients were determined by QPCR (n = 9). Expression data are shown following normalization for GAPDH. Data was analyzed by paired student T test. Two cases (in red) were subjected into western blot analysis.

Figure S4

A

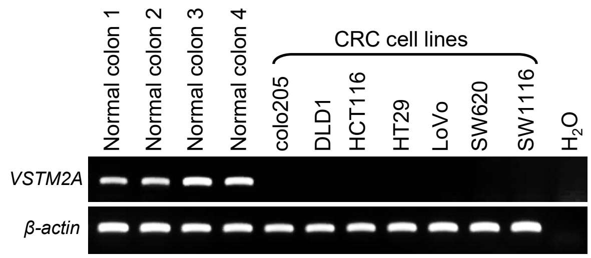

B

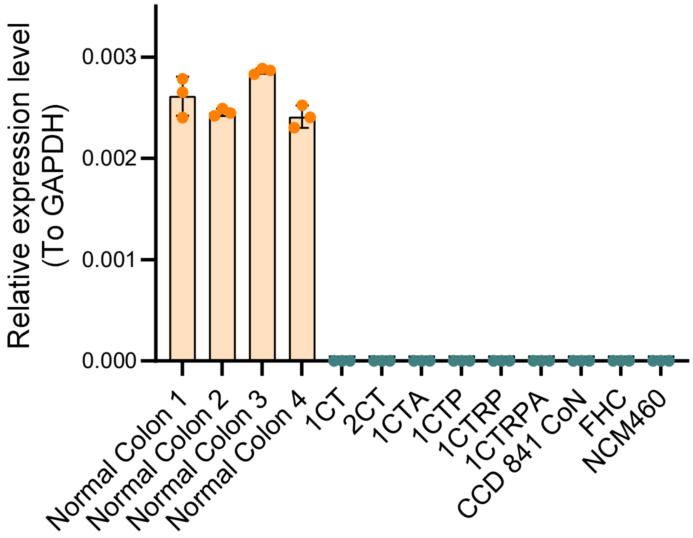

Cell line	*β-Actin (Ct)*	*VSTM2A (Ct)*
MC38	11.80	37.30
CT26	11.60	40.00
AGS	12.63	35.37
MKN28	16.03	39.40
SNU620	14.37	40.00
BGC823	16.42	40.00
MKN45	13.17	33.51
SNU638	15.49	36.84
MGC803	14.32	40.00
SNU668	13.76	37.98
TMK1	18.58	34.81

Figure S4. (A) Semi-PCR analysis of VSTM2A expression in normal colon tissues and CRC 2D cell lines. **(B)** QPCR analysis of transform and non-transformed human epithelial cells and murine CRC cell lines. Expression data are shown following normalization for GAPDH or β-Actin.

Figure S5

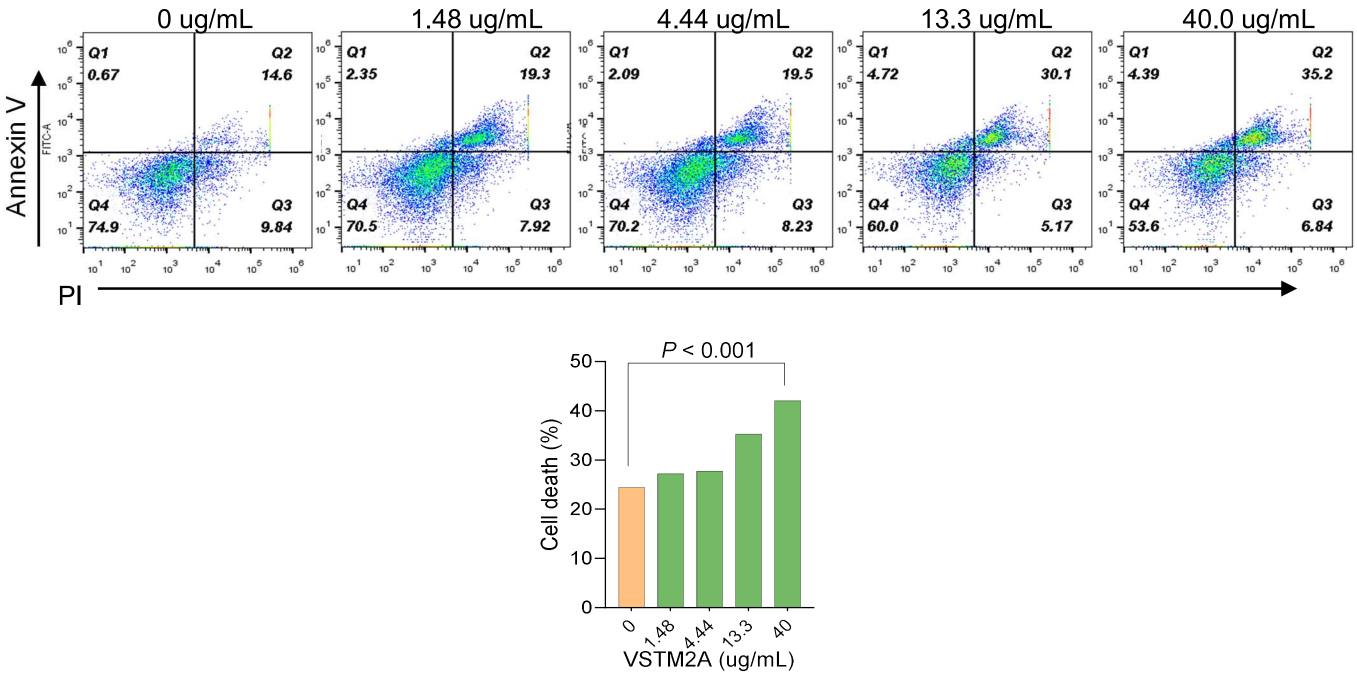

Figure S5. Cell apoptosis of PBMCs treated with various dose of recombinant human VSTM2A protein was quantified by flow cytometer.

Figure S6

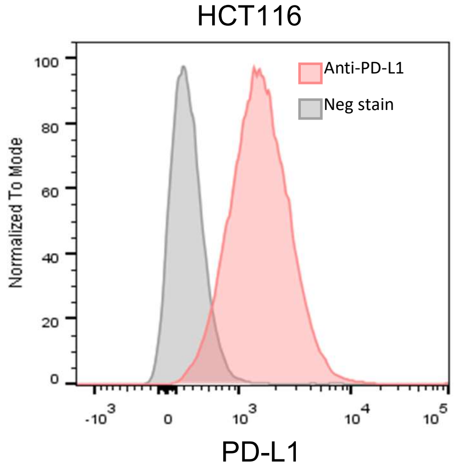

Figure S6. Cell surface expression of PD-L1 on HCT116 was determined by flow cytometry.

Figure S7

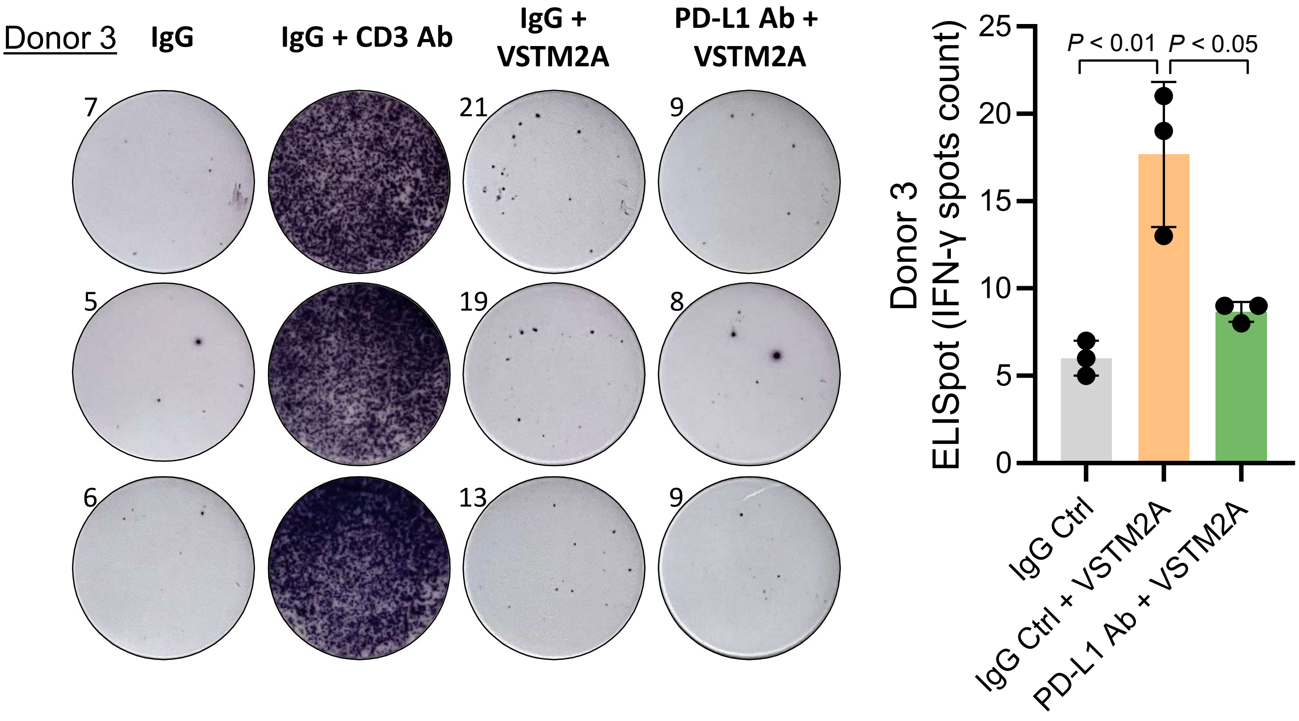

Figure S7. Images of the ELISpot assay showed the IFN-γ secretion by PBMCs (Donor 3) following 24 hours stimulation with the anti-CD3 antibody, Durvalumab (anti-PD-L1 immune checkpoint inhibitor) in the presence or absence of VSTM2A recombinant protein (1 ug/mL). Summary of the IFN-γ spot number is showed in the right panel.

Table S1. Correlation of VSTM2A expression with clinicopathologic parameters in 208 CRC patients.

Variable	VSTM2A expression				
	**Low (n=116)**	**%**	**High (n=92)**	**%**	***P*-value**
**Gender**					
Female	53	46.49	36	39.13	0.3234
Male	61	53.51	56	60.87	
**Age***					
≤60	33	28.95	28	30.43	0.8784
>60	81	71.05	64	69.57	
**AJCC stage***					
I/II	36	31.58	45	48.91	**0.0146**
III/IV	78	68.42	47	51.09	
**CD8a**					
High	37	31.90	52	56.52	**0.0004**
Low	79	68.10	40	43.48	

* 208 Case in the TMA in total with 2 cases missing of Gender, Age and AJCC stages information.

Table S2. Antibodies and QPCR primers used in this study.

Target	Supplier	Catalogue
Anti-human/mouse VSTM2A	Sigma-Aldrich	HPA024134
Anti-human GAPDH	ThermoFisher Scientific	PA585074
Anti-human PD-L1	R&D systems	AF156
Anti-human Phospho-LRP6	Cell Signaling Technology	2568P
Anti-Flag	Sigma-Aldrich	F1804
Anti-human CD8a	BioLedend	372902
Anti-human PD-L1 APC	BioLegend	329707
Anti-mouse CD8a	ThermoFisher Scientific	14008182
Anti-mouse CD45 FITC	BD Biosciences	553080
Anti-mouse CD3 PerCP-Cy5.5	BD Biosciences	560527
Anti-mouse CD45 BV510	BioLegend	103137
Anti-mouse CD8 PerCP-Cy5.5	BioLegend	100733
Anti-mouse CD3 PE	BioLegend	100206

Target	Sequence
Human-VSTM2A-QF	GGACCAAGATCAGCACAGTGAA
Human-VSTM2A-QR	TCATCCTTTTTCCTCACTTTGGA
Human-GAPDH-QF	GCACCACCAACTGCTTAGCA
Human-GAPDH-QR	TCTTCTGGGTGGCAGTGATG
Human-ACTB-QF	GTCTTCCCCTCCATCGTG
Human-ACTB-QR	AGGGTGAGGATGCCTCTCTT
Ms-Vstm2a-QF	ATGTGGCTGCAAGACACGAA
Ms-Vstm2a-QR	GGCTGGAGGTGGAGTGCAT
Ms-Actin-QF	GGCTGTATTCCCCTCCATCG
Ms-Actin-QR	CCAGTTGGTAACAATGCCATG
